# Serine residues 726 and 780 have nonredundant roles regulating STAT5a activity in luminal breast cancer

**DOI:** 10.1038/s41598-021-92830-8

**Published:** 2021-06-29

**Authors:** Alicia E. Woock, Jacqueline M. Grible, Amy L. Olex, J. Chuck Harrell, Patricija Zot, Michael Idowu, Charles V. Clevenger

**Affiliations:** 1 Department of Pathology, Virginia Commonwealth University, 1101 E. Marshall St, Sanger 4-006A, Richmond, VA 23298-06629 USA; 2 C. Kenneth and Dianne Wright Center for Clinical and Translational Research, Virginia Commonwealth University, Richmond, VA 23298 USA

**Keywords:** Breast cancer, Hormone receptors, Mechanisms of disease, Molecular biology, Transcription, Transcriptomics

## Abstract

In breast cancer, prolactin-induced activation of the transcription factor STAT5a results from the phosphorylation of STAT5a tyrosine residue 694. However, its role in mammary oncogenesis remains an unsettled debate as STAT5a exhibits functional dichotomy with both pro-differentiative and pro-proliferative target genes. Phosphorylation of STAT5a serine residues, S726 and S780, may regulate STAT5a in such a way to underlie this duality. Given hematopoiesis studies showing phospho-serine STAT5a as necessary for transformation, we hypothesized that serine phosphorylation regulates STAT5a activity to contribute to its role in mammary oncogenesis, specifically in luminal breast cancer. Here, phosphorylation of S726-, S780-, and Y694-STAT5a in response to prolactin in MCF7 luminal breast cancer cells was investigated with STAT5a knockdown and rescue with Y694F-, S726A-, or S780A-STAT5a, where the phospho-sites were mutated. RNA-sequencing and subsequent Ingenuity Pathway Analysis predicted that loss of each phospho-site differentially affected both prolactin-induced gene expression as well as functional pathways of breast cancer (e.g. cell survival, proliferation, and colony formation). In vitro studies of anchorage-independent growth and proliferation confirmed distinct phenotypes: whereas S780A-STAT5a decreased clonogenicity, S726A-STAT5a decreased proliferation in response to prolactin compared to wild type STAT5a. Collectively, these studies provide novel insights into STAT5a activation in breast cancer pathogenesis.

## Introduction

In mammary epithelium, the polypeptide prolactin (PRL) acts through its cognate receptor (PRLr), and is responsible for terminal maturation and differentiation of lactating glands during pregnancy^1–7^. As a potent mitogenic factor, PRL/PRLr signaling is also associated with mammary tumorigenesis^2,8–12^. Canonical PRL/PRLr signaling involves the tyrosine janus kinase 2 (JAK2), which phosphorylates the transcription factor (TF) signal transducer and activator of transcription 5a (STAT5a) on tyrosine 694 (Y694). The resulting phosphorylated STAT5a (pY694-STAT5a) dimerizes and translocates to the nucleus.

STAT5a is one of seven members of the STAT family of transcription factors. The STAT family is highly conserved, each having a tyrosine residue near residue position 700 that is phosphorylated by JAK in the cytoplasm. This phosphorylation event has historically been considered as the activation switch for STAT (pY-STAT), whereas dormant, unphosphorylated STAT (upY-STAT), has long been considered to have no significant functions. However, accumulating evidence shows that upY-STATs are present in the nucleus regardless of cytokine stimulation^13,14^. Studies have illustrated that upY-STATs actively bind chromatin to drive gene expression distinct from that of the pY-STAT forms^13,15–20^. Notably, other functions described of upY-STATs include promotion of heterochromatin and global downregulation of transcription^14,21^. These differing reports highlight the possibility of stimulus- and tissue-specific functions of upY-STAT vs. pY-STAT.

Compounding this growing understanding of the regulation of STAT activity, STATs (with the exception of STATs 2 and 6) also have serine residues in the Transactivation Domain (TAD) that are capable of being phosphorylated, with known function in regulating STAT activity^22–27^. To this point, STAT5a has two serine residues, S726 and S780, which regulate STAT5a-dependent malignant hematopoietic transformation^28,29^. The function of phosphorylation of either S726 or S780 in PRL-responsive normal or malignant breast tissue, however, is less well understood.

Several studies regarding STAT5a serine phosphorylation have focused on PRL-induced β-casein expression using luciferase gene reporter assays^30–32^. However, the results of these studies are inconsistent regarding the loss of the serine residues on PRL-stimulated β-casein gene induction. Beuvink et al. reported that loss of S726 or S780 had no effect on PRL-stimulated β-casein promoter activity^31^. While Yamashita et al. also observed that loss of S780 had no effect on PRL-stimulated β-casein promoter activity, in contrast they found that expression of phospho-deficient S726A-STAT5a in COS-7 and MCF7 cells increased the PRL-stimulated β-casein promoter activity compared to (wild type) WT-STAT5a^30,32^. These studies did not examine the physiologic effect of phospho-deficient STAT5a expression.

In this study, we examine the expression of serine-phosphorylated (pS-)STAT5a in human breast cancer, describe the effects of tyrosine and serine phospho-deficient STAT5a mutants on breast cancer characteristics in vitro*,* and show for the first time how these residues differentially affect transcriptional programs in luminal breast cancer.

## Results

### Stat5a serine residues 726 and 780 are phosphorylated in human breast cancer cell lines and patient tumor samples

**Figure 1:**
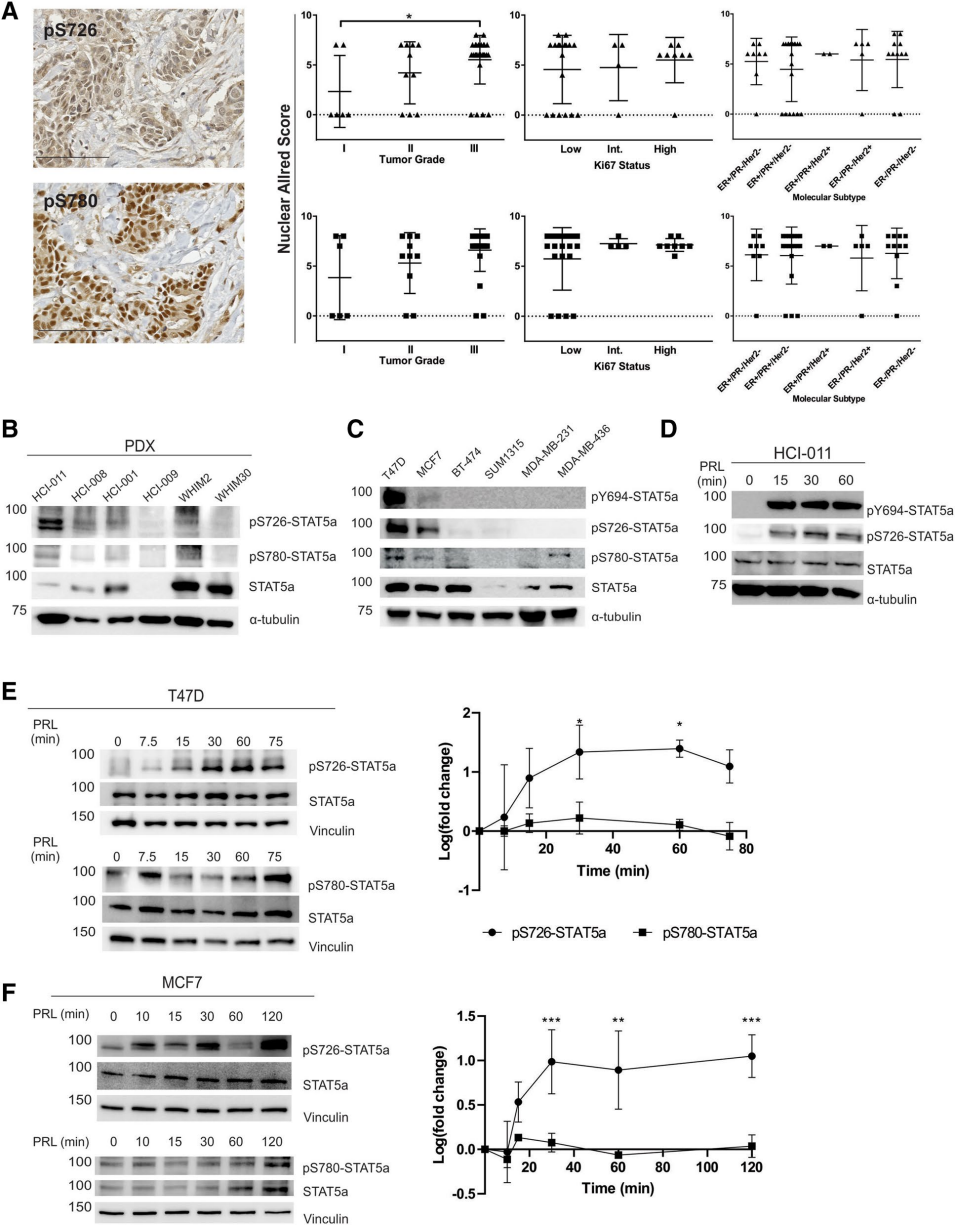
STAT5a S726 and S780 phosphorylation occur in human breast cancer. (**A**) Representative anti-pS726- and anti-pS780-STAT5a IHC images from unmatched grade III, ER-/PR-/Her2 amplified primary tumors included in the TMA (scale bars, 100 μm). Note the nuclear accumulation of pS726- and pS780-STAT5a. Graphs show quantification of nuclear Allred scores according to the tumor grade, Ki67 status, and molecular subtypes. Panels of PDX samples (**B**) and established breast cancer cell lines (**C**) immunoblotted for pY694-, pS726- and pS780-STAT5a. Note the presence of pS726/S780-STAT5a in the luminal B subtype (ER+/PR+/Her2−) sample HCI-011, and in the luminal B cell lines T47D and MCF7, along with pY694-STAT5a. Lysates for (**C**) were run in triplicate and representative blots for STAT5a and tubulin are shown. (**D**) HCI-011 lysates were subjected to PRL stimulation and analysis for pS726-STAT5a and pY694-STAT5a induction. PRL stimulation of the human breast cancer cell lines T47D (**E**), and MCF7 (**F**), show induction of pS726-STAT5a but constitutive pS780-STAT5a, quantified by densitometric analysis. n = 3, *p ≤ 0.03, **p = 0.001, ***p ≤ 0.0004 compared to untreated, time 0. Graphs and statistics generated in GraphPad Prism 9.0.0 for Windows (GraphPad Software, San Diego, California USA, www.graphpad.com).

Serine phosphorylation of STAT5a has been observed in mouse mammary epithelial cells throughout mammary development, however this has yet to be clinically characterized in human breast cancer^31,32^. We performed immunohistochemical (IHC) staining of a breast cancer tissue microarray (TMA) using phospho-specific antibodies for pY694-, pS726-, and pS780-STAT5a, as well as total STAT5a. The TMA consisted of 47 clinically staged cancers I-III, with diagnoses ranging from ductal carcinoma in situ (DCIS) to invasive ductal or lobular carcinoma (Supplemental Table S1). Visual scoring of STAT5a S726 phosphorylation revealed a significant increase in nuclear intensity in tumor samples grade III compared to grade I (nuclear Allred scores of 6 and 2, respectively; Fig. 1A). Notably, STAT5a S780 phosphorylation was also observed in the nucleus of tissue samples, however there was no significant association of expression with either tumor grade or proliferative status (Ki67 staining; Fig. 1A). While total nuclear STAT5a expression remained high throughout malignant progression in these breast tissue samples, a weak signal for nuclear pY694-STAT5a was observed in the tissue samples independent of tumor grade, hormone receptor status, or Ki67 staining, consistent with previous reports (Supplemental Figure S1)^33^.

Patient derived xenograft (PDX) whole cell extract (WCE) samples (Fig. 1B) and established breast cancer cell lines (Fig. 1C, Supplemental Table S2) spanning the intrinsic molecular subtypes were probed for STAT5a S726 and S780 phosphorylation^34^. HCI-011 (luminal B, ER+/PR+/HER2−) showed expression of both pS726- and pS780-STAT5a (Fig. 1B), in concordance with the luminal A/B breast cancer cell lines MCF7 and T47D (Fig. 1C)^35^. Interestingly, the claudin-low breast cancer cell line MDA-MB-436 exclusively exhibited pS780-STAT5a as well (Fig. 1C)^36^. Ex vivo analysis of HCI-011 lysates demonstrated pY694- and pS726-STAT5a expression following PRL stimulation (Fig. 1D). Likewise, PRL stimulation of both T47D cells and MCF7 cells results in robust S726-STAT5a phosphorylation (Fig. 1E,F). However, phosphorylation of STAT5a at S780 was observed to be constitutive and independent of PRL stimulation, consistent with literature findings (Fig. 1E,F)^31,32^.

### STAT5a serine residues have nonredundant transcriptional roles in luminal breast cancer cells

**Figure 2:**
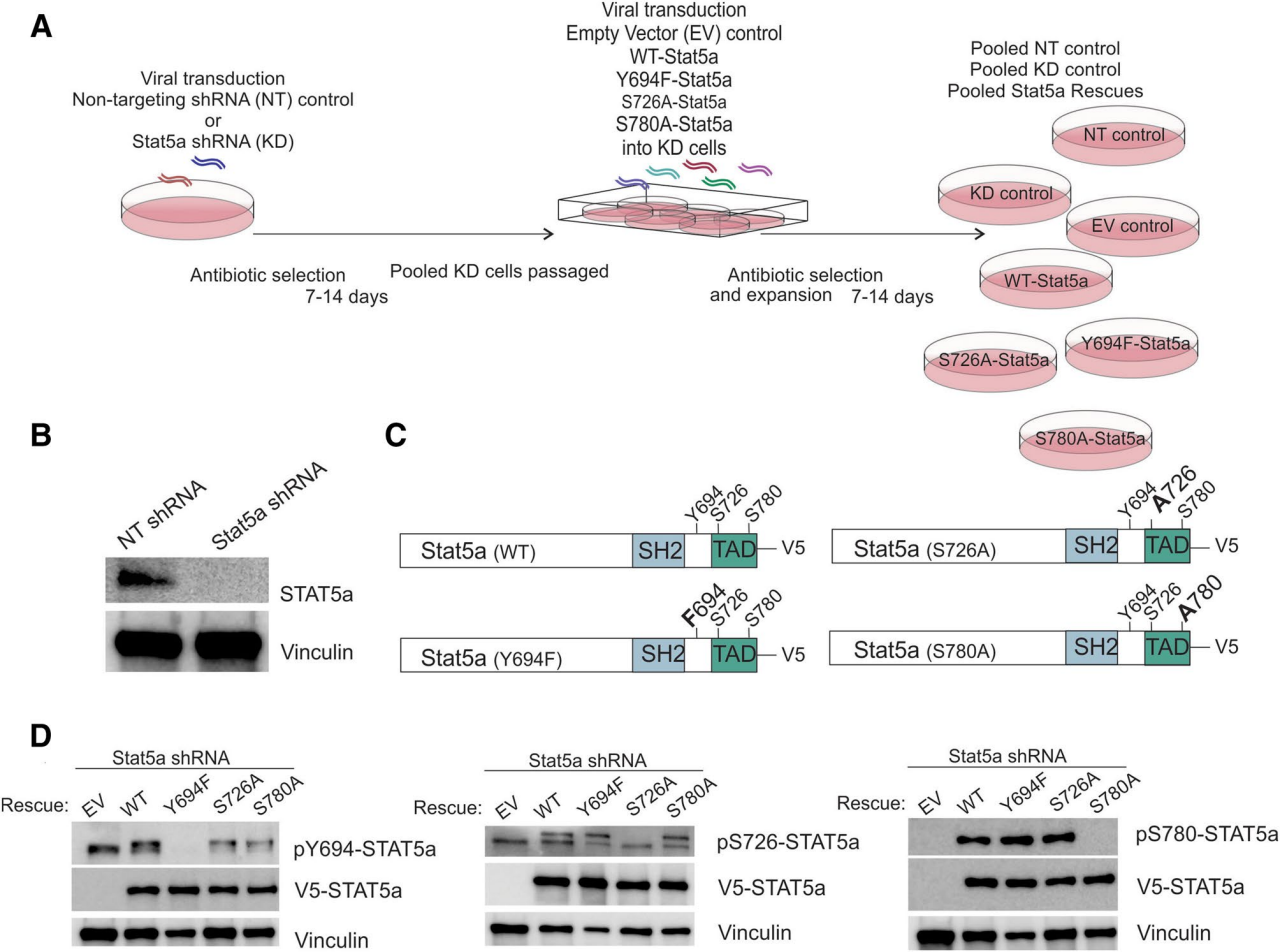
The human breast cancer cell line MCF7 as a model for STAT5a serine phosphorylation. (**A**) Schema for generating of stable knockdown and rescue cell lines generated in CorelDraw Graphics Suite X7 (Version 17.0.0.491, www.corel.com). (**B**) STAT5a knockdown in MCF7 cells after antibiotic selection confirmed by WB. (**C**) Representations of WT-, Y694F-, S726A-, and S780A-STAT5a constructs with C-terminal V5 tags used to generate MCF7 rescue cell lines. Confirmation of STAT5a rescue expression in MCF7 cells by (**D**) protein analysis with appropriate antibodies targeting each phospho-site of STAT5a.

Previous studies have shown that STAT5a serine phosphorylation affects PRL-induced STAT5a DNA binding kinetics (as measured by electrophoretic mobility shift assay, or EMSA) and phosphorylation kinetics of residue Y694, however the role of these phospho-sites in PRL-regulated gene expression has not been investigated on a global scale^31^. We hypothesized that expression of serine phospho-deficient STAT5a mutants would affect breast cancer transcriptomic programs and these alterations in gene expression patterns would lead to changes on a phenotypic scale. To test this hypothesis, we utilized MCF7 cells stably transduced with either STAT5a shRNA (STAT5a KD) or nontargeting (NT) control shRNA (Fig. 2A,B). Pools of MCF7 cells with stable STAT5a KD were rescued with lentiviral transduction of WT-STAT5a, phospho-deficient single-point mutants (containing a C-terminal V5-tag), or empty vector (EV), such that stable novel MCF7 cell lines were established. Hereafter, these transfectants are referred to as WT-STAT5a, Y694F-STAT5a, S726A-STAT5a, S780A-STAT5a, or EV (Fig. 2A,C). Relative STAT5a KD and rescue protein expression was confirmed by Western blot (WB; Fig. 2B–D). Endogenous STAT5a KD was determined as being ~ 50% effective by qRT-PCR, and rescue STAT5a transcript expression was 250–400-fold higher than that of the nontargeting control (Supplemental Figure S2). However, on the protein level, STAT5a was equally expressed by the rescues (Fig. 2D). Therefore, quantification of all subsequent studies using these MCF7 pooled rescue cell lines is reported normalized to V5-STAT5a expression.

**Figure 3:**
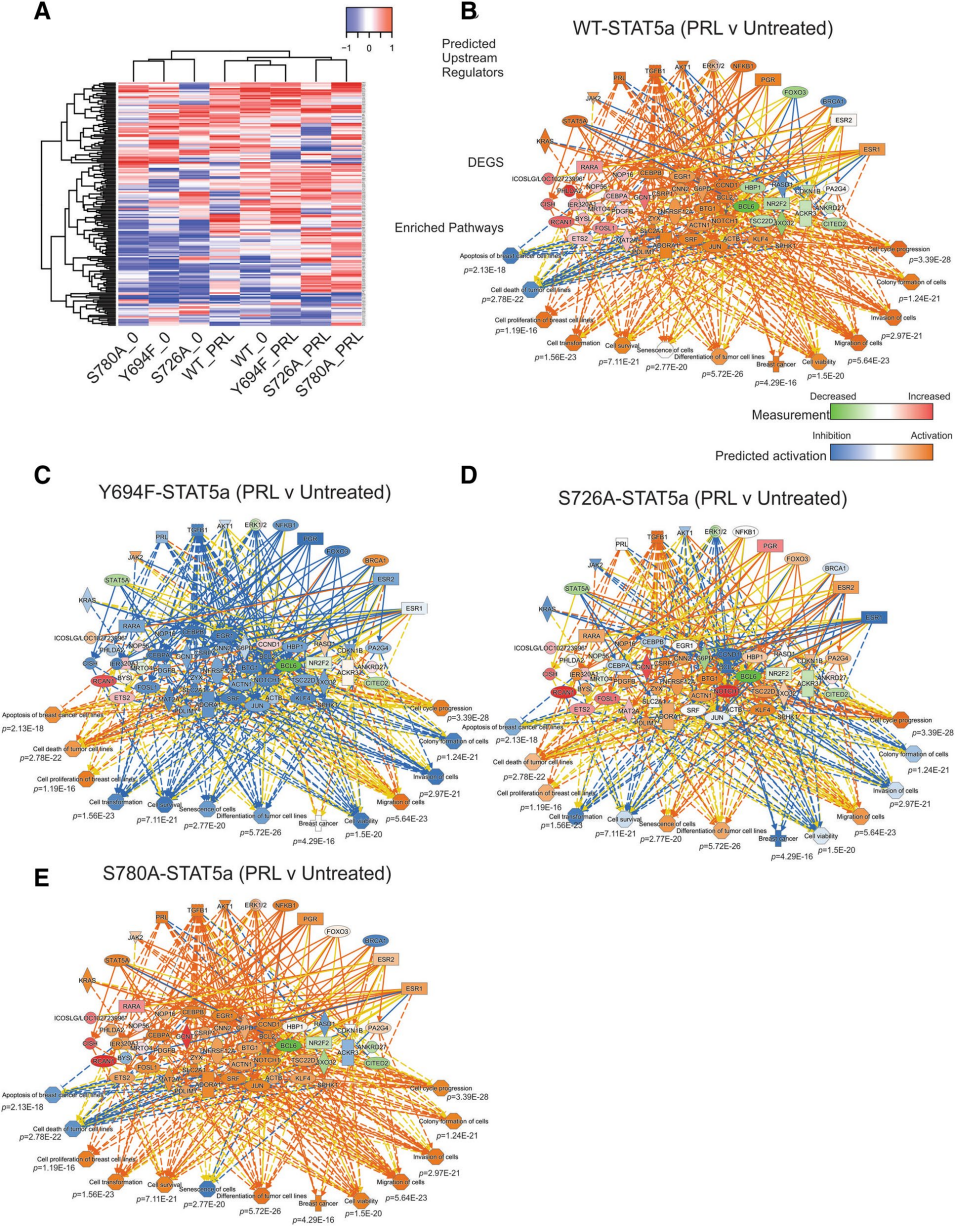
Expression of STAT5a phospho-mutants differentially affects PRL-regulated gene expression in MCF7 cells. (**A**) Supervised clustering of each STAT5a species’ top 50 DEGs (≥ 1.5-fold change) from RNA-seq analysis of PRL-treated versus untreated MCF7 cells expressing STAT5a rescues using the heatmap2 tool on www.usegalaxy.org (Galaxy Version 3.0.1). (**B**) IPA functional pathway enrichment analysis shows upstream regulators and predicted pathway activation or inhibition with PRL treatment of WT-STAT5a-expressing MCF7 cells. (**C**–**E**) IPA functional pathway analysis of indicated STAT5a phospho-mutants overlaid with the regulatory network shown in panel B to predict activation or inhibition. *p* value calculated by Fisher’s exact test for networks generated in IPA with predicted effects based on the similarity of gene expression values to annotated network relationships in the Ingenuity knowledge base (IPA software 2020 Version 62,089,861, Qiagen).

These generated stable MCF7 cells were then stimulated with PRL (250 ng/mL) and subjected to RNA sequencing (RNA-seq), to assess global transcriptomic changes following STAT5a mutagenesis. These analyses revealed that following PRL treatment, MCF7-WT-STAT5a cells had 510 differentially expressed genes (DEGs), and each STAT5a phospho-deficient mutant had fewer DEGs than the WT-STAT5a [ranging from 171 (Y694F-STAT5a) to 285 DEGs (S726A-STAT5a); Supplemental Table S3]. Next, the top 50 DEGs from the four STAT5a rescues when treated with PRL vs untreated (for a total of ~ 200 DEGs) were subjected to supervised clustering (Fig. 3A). Results from Y694F-STAT5a MCF7 cells treated with PRL were most similar to untreated WT-STAT5a MCF7 cells, indicating that loss of Y694-STAT5a is sufficient to inhibit most PRL-induced transcript expression. Hierarchical clustering of all significant DEGs supported this finding (Supplemental Figure S3). When treated with PRL, S726A- and S780A-STAT5a MCF7 cells had similar expression of the top 50 DEGs from each contrast, visualized by the clustering of the S726A + PRL and S780A + PRL samples. Interestingly, when untreated, the three STAT5a point mutants top 50 DEGs cluster away from the untreated WT-STAT5a MCF7 cells. This indicates that in these cells STAT5a may drive expression of a subset of genes in the absence of tyrosine or serine phosphorylation. This would be a distinct function for STAT5a that is independent from its role of inducible, phosphorylation-dependent gene expression, similar to descriptions of STAT3^17,37^.

WT-STAT5a PRL-induced or inhibited genes were compared to previous PRL-transcriptomic data released by our laboratory, such that after matching the criteria that the DEG be present in at least two datasets, 105 putative PRL-regulated genes were identified (Supplemental Table S4)^38,39^. A subset of these genes were used to identify functionally-enriched pathways with PRL treatment that may be changed by expression of the STAT5a mutants through Ingenuity Pathway Analysis (IPA, Qiagen, Fig. 3B). The PRL treatment of WT-STAT5a MCF7 cells significantly activated pathways responsible for cellular proliferation and survival, colony formation, migration, and invasion. Interestingly, expression of the Y694F-STAT5a mutant had an opposite effect on 10 of the 13 (76.9%) queried functional predicted pathways compared to WT-STAT5a, including cell survival and colony formation pathways (Fig. 3C). Expression of the S726A-STAT5a mutant similarly affected 7 of the same pathways that Y694F-STAT5a expression did (7/13, 53.8%), and S780A-STAT5a affected the senescence pathway only (1/13, 7.7%; Fig. 3D,E).

Confirmation of the gene expression changes observed in the RNA-seq data was done with MCF7 cells similarly stimulated with PRL. qRT-PCR was performed on a panel of genes, including the gene encoding cytokine-inducible SH2 containing protein (*CISH*), a known STAT5a-target gene induced by PRL in breast epithelial cells and in mammary carcinoma^40,41^. As seen in Supplemental Figure S4, loss of pY694 completely ablated PRL-induced *CISH* expression compared to WT-STAT5a. S726 and S780 point mutagenesis similarly were sufficient to significantly reduce *CISH* expression following PRL stimulation, compared to that of WT-STAT5a, albeit to a lesser extent than was observed for the tyrosine mutant. These results indicate some role for serine phosphorylation in modulating STAT5a transcriptional activation. Further, Estrogen Receptor α (*ESR1*) expression was shown to rely on phosphorylation of the phospho-serine residues differentially. Loss of pS726 increased PRL-induced *ESR1* expression relative to WT-STAT5a (an increase that is more pronounced when the expression of total STAT5a is considered; Supplemental Figure S4), whereas loss of pS780 did not significantly affect *ESR1* expression.

### Functions of the STAT5a phospho-serine residues in vitro are independent of pY694

**Figure 4:**
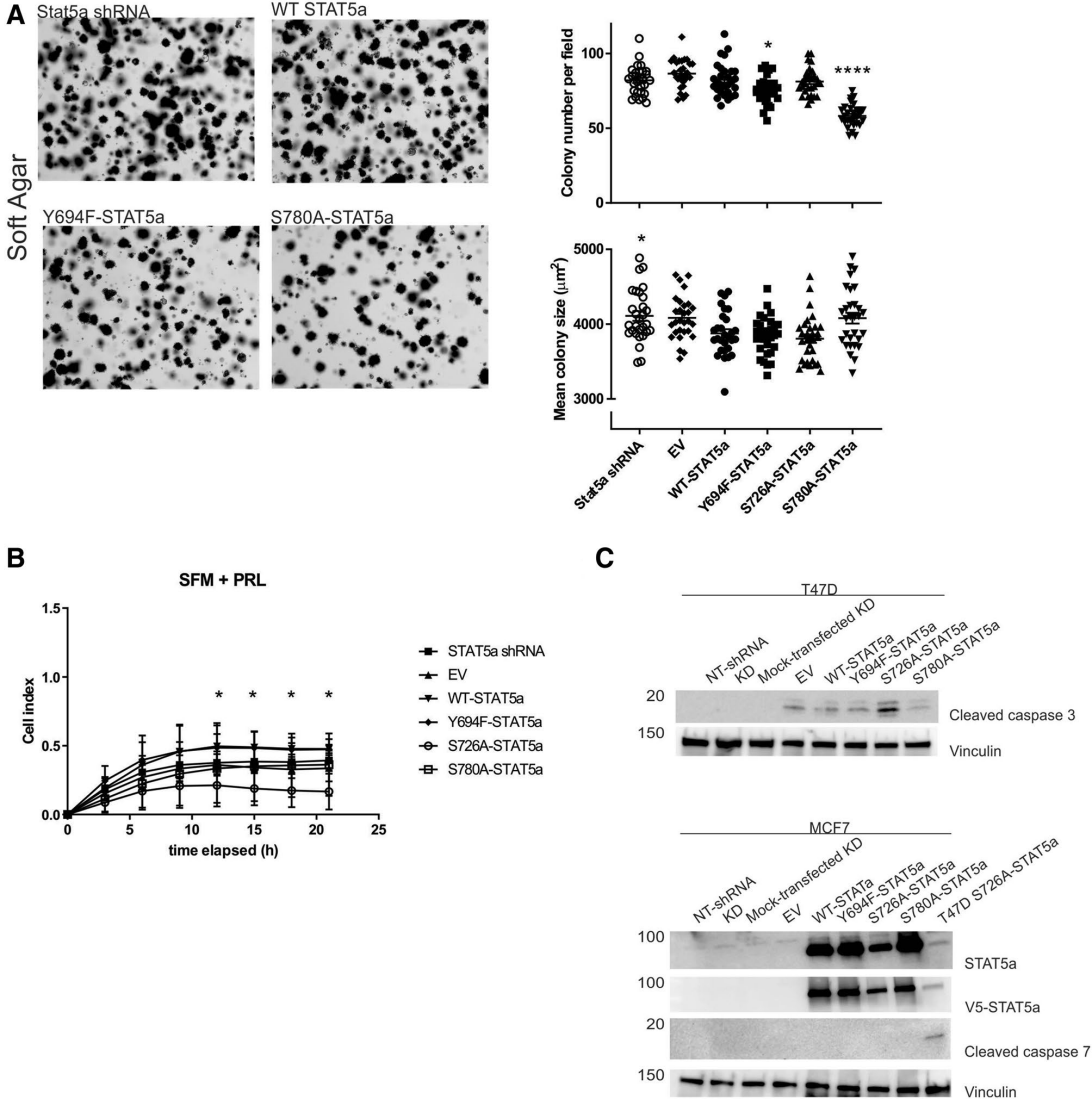
Effect of single-point phospho-deficient STAT5a mutants on characteristics of MCF7 cells. (**A**) Representative images and quantification of number and size of the colonies formed in soft agar by indicated MCF7 cells with knockdown (KD) of STAT5a rescued by phospho-deficient mutants. n = 3, *p < 0.02 compared to WT-STAT5a. (**B**) Proliferation in serum-free media (SFM) supplemented with PRL was assessed using XCELLigence, cell index indicates proliferation. n = 3, *p* < 0.02 compared to WT-STAT5a. (**C**) Immunoblots of T47D cells (top) and MCF7 (bottom) cells carrying transient rescues of STAT5a point mutants assessed for apoptosis by expression of cleaved caspase 3 or 7. Graphs and statistics generated in GraphPad Prism 9.0.0 for Windows (GraphPad Software, San Diego, California USA, www.graphpad.com).

To confirm these functional predicted effects observed with expression of these STAT5a mutants, we next assessed the loss of the STAT5a phospho-sites on cancer characteristics in vitro. The ability of transformed cells to grow independently and survive without extracellular matrix attachment can be used to understand cell differentiation, transformation and tumorigenesis^42,43^. Therefore, we performed a quantitative evaluation of soft agar assay with various growth conditions. In quantifying three independent experiments compared to WT-STAT5a rescue colonies, MCF7 cells rescued with either Y694F- or S780A-STAT5a had fewer colonies (Fig. 4A, Supplemental Figure S5). This indicates that not only phosphorylation of Y694, but also phosphorylation of S780 on STAT5a is involved in the clonogenicity of breast cancer, in that fewer MCF7 colonies were established with loss of the phospho-sites. When analyzed for the size of colonies established, total STAT5a knockdown had increased colony size compared to WT-STAT5a rescue (*p* = 0.02, Fig. 4A). Together, these results illustrate that STAT5a expression restricts the extent of breast cancer outgrowth and clonogenicity, and that loss of any individual phospho-regulatory site on STAT5a does not mimic total loss of STAT5a^44^.

To further study the roles of each phospho-site of STAT5a, proliferation in two-dimensional (2D) culture of the MCF7 rescues was measured on an xCELLigence apparatus (Agilent Technologies). In the presence of PRL, the S726A-STAT5a rescues showed decreased proliferation compared to WT-STAT5a rescues from 12 to 21 h (Fig. 4B). This change from WT-STAT5a indicates a role for the phosphorylation of STAT5a on S726 mediating PRL-induced proliferation.

The migratory ability of MCF7 cells in 2D culture was analyzed by real-time imaging using a scratch wound assay. 72 h after establishing a wound in a confluent cell layer, all cell lines containing STAT5a rescues were 100% confluent once more (Supplemental Figure S5). Unremarkably, the knockdown of STAT5a, or the rescue with various phospho-deficient point mutations, had no effect on the ability of MCF7 cells to migrate on the 2D plastic layer, indicating single-phospho-point mutants are not sufficient to elicit a phenotypic effect on migration.

STAT5a and PRL are both implicated in dysregulating apoptotic machinery in breast cancer cells, which was confirmed in our pathway analysis of the MCF7 STAT5a mutants RNA-seq data (Fig. 3B–E)^24,45–47^. Further, it was observed that the T47D breast cancer cell line, when either transiently transfected or stably transduced with the STAT5a rescue constructs, had variable success in establishing 2D colonies; ultimately only transient expression was viable for performing quantitative assays. MCF7 cells do not express caspase 3, therefore to investigate this phenomenon, we analyzed protein expression of cleaved caspases 3 (CC3) and 7 (CC7) in T47D and MCF7 cells, respectively, with endogenous STAT5a knockdown and transient re-expression of the STAT5a rescues (Fig. 4C)^48,49^. T47D cells rescued with S726A-STAT5a were highly positive for CC3, indicating that this serine residue contributes to the survival of the T47D cell line. On the other hand, MCF7 cells exhibited no CC7, indicating that these cells do not rely on STAT5a for survival. This difference in STAT5a regulation of apoptosis may be due to the difference in apoptotic pathways used in T47D and MCF7 cells, or by the reported reliance of T47D cells on STAT5a for survival, and now, specifically pS726-STAT5a^50^. Overall, these studies illustrate that phosphorylation of S726 may be responsible for STAT5a involvement in evasion of apoptosis, while phosphorylation of S780 is responsible for STAT5a involvement in tumor clonogenicity and establishment of anchorage-independent colonies.

### The canonical pathway of STAT5a is independent of phosphorylation of S726 or S780

**Figure 5:**
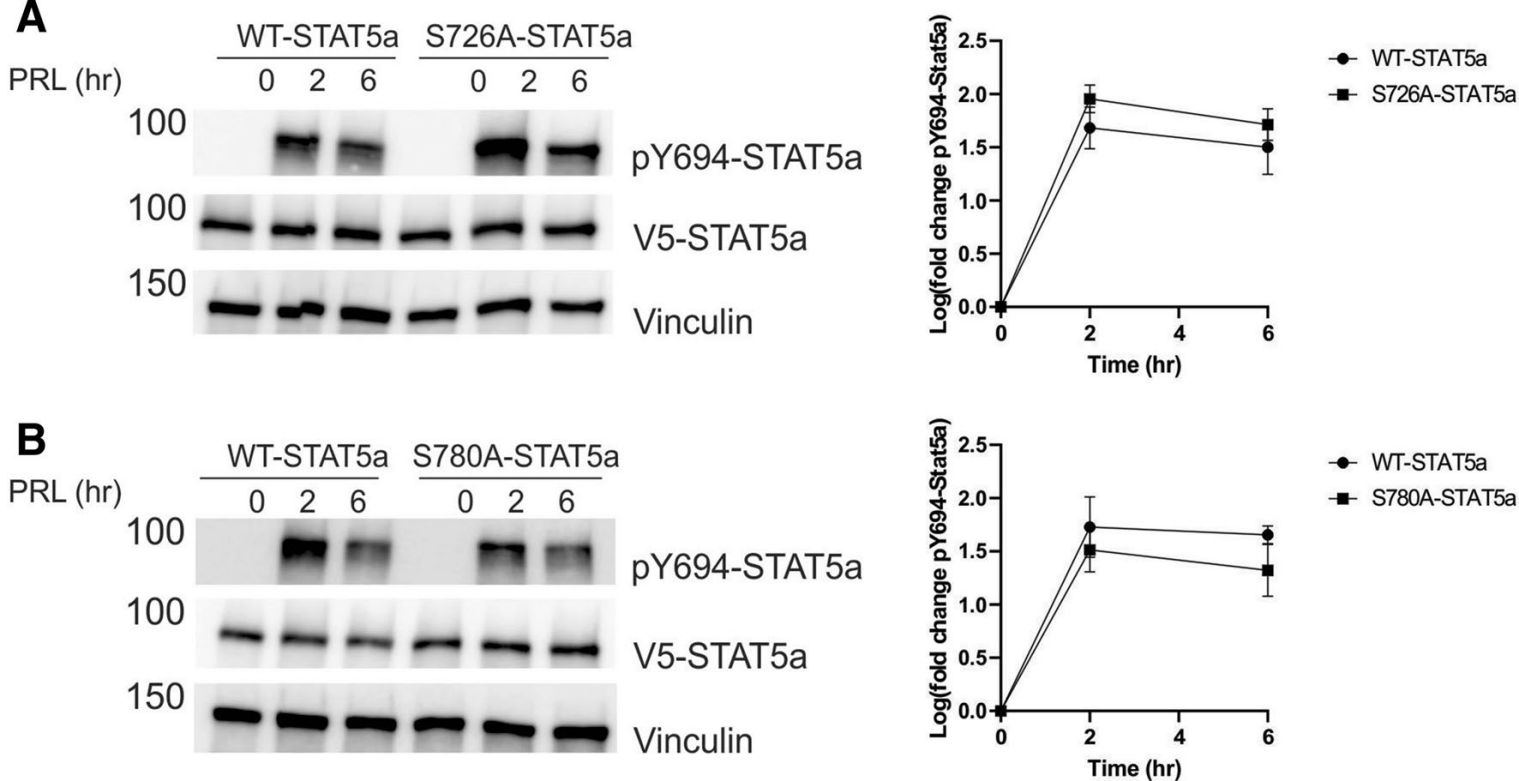
Induction of phosphorylation on residue Y694 is independent of either S726 or S780. MCF7 cells with stable re-expression of either WT-STAT5a, S726A-STAT5a (**A**), or S780A-STAT5a (**B**) were treated with PRL up to 6 h. Representative blots of 3 independent experiments for pY694-STAT5a induction shown, with quantification by densitometric analysis performed in GraphPad Prism 9.0.0 for Windows (GraphPad Software, San Diego, California USA, www.graphpad.com).

To determine the role of pS726 and pS780 in regulation of the canonical pathway of STAT5a signaling, the kinetics of phosphorylation of Y694-STAT5a was analyzed in the corresponding rescue cell lines. Previously it was reported that loss of S726 stabilized PRL-induction of pY694, while WT-STAT5a loses pY694 by 5 h of PRL stimulation^31^. However, MCF7 cells re-expressing either WT-STAT5a or S726A-STAT5a have comparable induction of pY694 up to 6 h (Fig. 5A). Similarly, we found that after 2 h and 6 h of PRL stimulation, the S780A-STAT5a mutant MCF7 cells had no effect on PRL-induced phosphorylation of the JAK2-target Y694 (Fig. 5B), indicating that neither phospho-serine contributes to maintenance of pY694.

### Unphosphorylated STAT5a undergoes nuclear translocation

**Figure 6:**
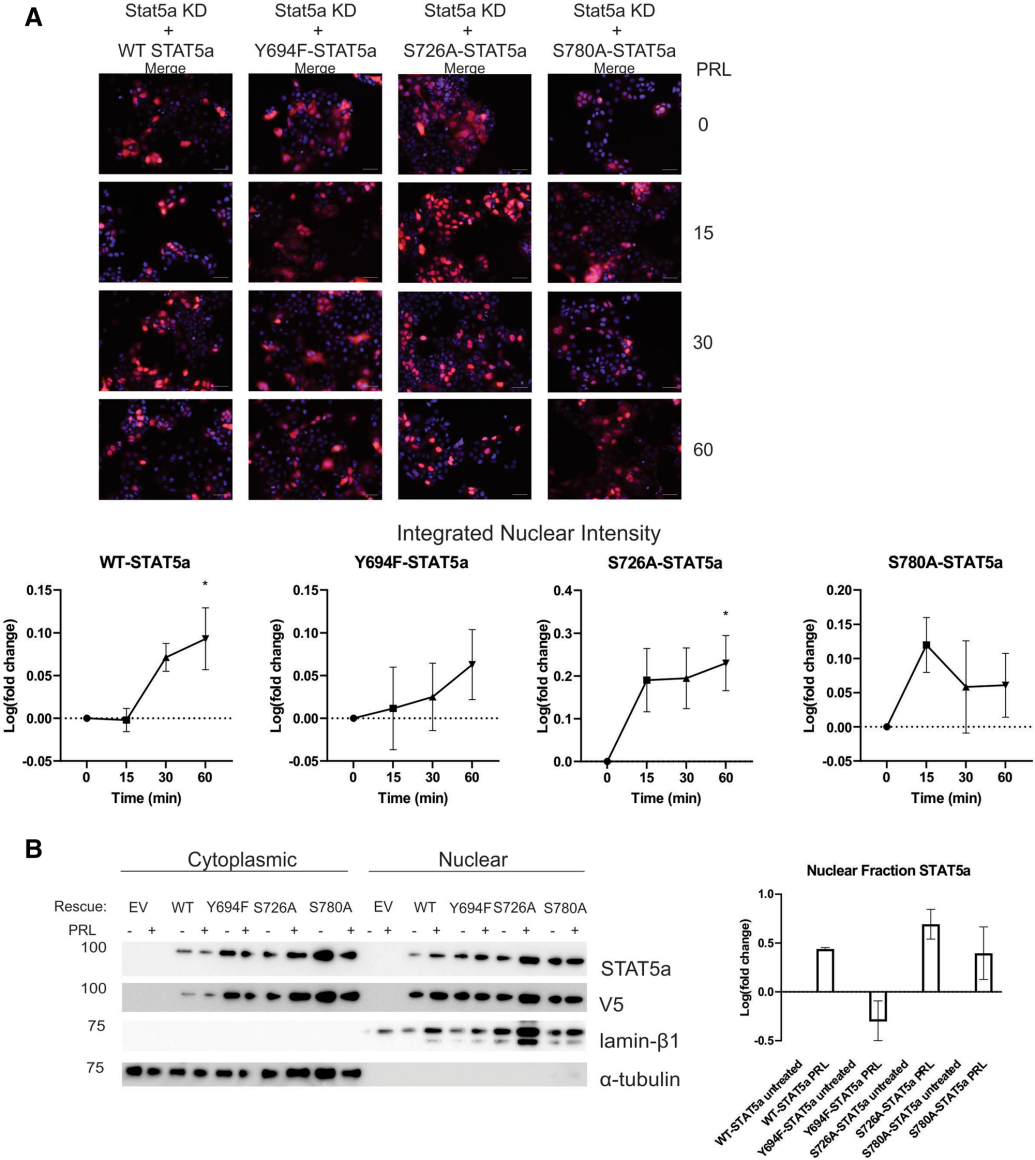
Nuclear translocation of STAT5a occurs in absence of pS726 and/or pS780. MCF7 cells with stable re-expression of STAT5a carrying specific phospho-deficient point mutations were imaged with immunofluorescence with up to 60 min PRL stimulation, representative merged Hoechst and STAT5a images shown, and quantified for nuclear translocation (**A**) or analyzed by WB nuclear cytoplasmic fractionation after 15 min PRL stimulation (**B**). *p < 0.04 compared to untreated, time 0. Graphs and statistics generated in GraphPad Prism 9.0.0 for Windows (GraphPad Software, San Diego, California USA, www.graphpad.com).

To determine if differences in transcriptional activity between WT-STAT5a and the phospho-site STAT5a mutants is due to altered nuclear localization of STAT5a, we performed both immunofluorescence (IF) imaging and WB analysis of nuclear-cytoplasmic fractionation with PRL stimulation (Fig. 6, Supplemental Figure S6). Nuclear-localized WT-STAT5a began to increase by 30 min and was significantly increased by 60 min of PRL stimulation. While there was an upward trend for Y694F-STAT5a translocating to the nucleus, there was a no net change in translocation into or out of the nucleus with PRL stimulation (Fig. 6A). The loss of S726 phosphorylation increased STAT5a nuclear translocation compared to WT over time with PRL stimulation (Fig. 6A) and retained STAT5a in the nucleus up to 60 min following PRL stimulation. The loss of S780 phosphorylation shifted the kinetics of STAT5a nuclear translocation such that the amount of STAT5a peaked in the nucleus with 15 min of PRL treatment, and then decreased to close to baseline after 30 min (Fig. 6A). WB analysis of nuclear and cytoplasmic fractions after 15 min of PRL treatment illustrates a snapshot of these trends (Fig. 6B). In agreement with previous data, Y694F-STAT5a was present in the nucleus independent of PRL stimulation, confirming reports that have also found upY694-STAT5a is continuously shuttled into and out of the nucleus (Fig. 6B)^13,14^. These results indicate that each phospho-serine could play an independent role in STAT5a nuclear translocation, degradation, or export.

**Figure 7:**
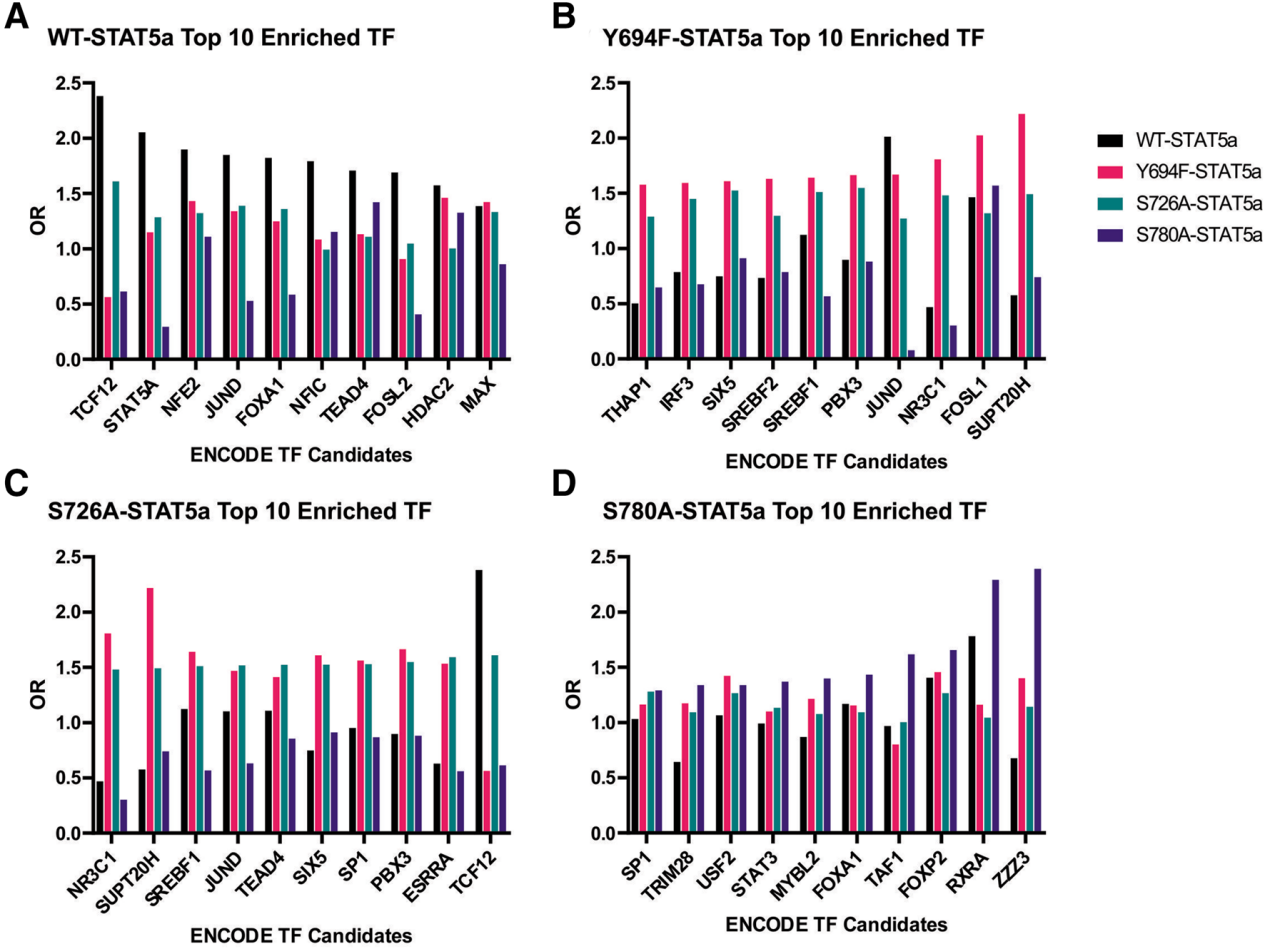
Transcription factor gene set analysis identifies complex transcriptional networks contributing to STAT5a mutant-driven phenotypes. TF analysis of the DEGs from MCF7 cells treated with PRL for (**A**) WT-STAT5a, (**B**) Y694F-STAT5a, (**C**) S726A-STAT5a, and (**D**) S780A-STAT5a. The ENCODE project ChIP-seq data set library was used to find the top-ranking transcription factors by odds ratio (OR), *p* < 0.05. Graphs generated in GraphPad Prism 9.0.0 for Windows (GraphPad Software, San Diego, California USA, www.graphpad.com).

In order to examine how STAT5a phospho-mutants regulate the functional phenotypes uncovered in this study, we used our RNA-seq data in combination with the open-source Enrichr web-based platform^51^. Enrichr transcriptional gene set analysis (using ENCODE project chromatin immunoprecipitation [ChIP]-seq data) confirmed STAT5a is one of the top transcriptional regulators in the WT-STAT5a MCF7 cells when treated with PRL (OR 2.05, *p* = 0.03; Fig. 7A), along with HDAC2, a class IIb histone deacetylase similar to HDAC6 (a known STAT5a cofactor)^52^. Further, amongst the top 10 enriched TF terms, the AP-1 subunits (JUN and FOS), as well as the FOX TF family (FOXA1), were also present, confirming our laboratory’s recent findings that STAT5a shares chromatin binding sites with these TF families as well as the ETS, AP-2, SP/KLF, CREB, and Nuclear Receptor (NR, e.g. ESR1) TF families^53^. Conversely, Enrichr analysis of Y694F-STAT5a or S780A-STAT5a data did not identify STAT5a as one of the top significant transcriptional regulators with PRL treatment (Fig. 7B). Furthermore, the STAT5a transcriptional signature with PRL treatment was significantly enriched in the case of S726A-STAT5a Enrichr analysis (OR 1.21, *p* = 0.007). Intriguingly, expression of Y694F- or S726A-STAT5a increased the involvement of the SREBF [Sterol regulatory element-binding factor, or protein (SREBP)] TF family (Fig. 7B,C), that was not seen with the expression of S780A-STAT5a (Fig. 7D). Expression of S780A-STAT5a with PRL treatment, however, showed involvement of STAT3, SP1, and two of the FOX TF family members (Fig. 7D). Overall, these data indicate that various transcription factor families may serve to compensate for the loss of pY694- and pS-STAT5a differentially to uphold oncogenic signals in MCF7 cells.

## Discussion

The phospho-regulation of STAT5a by PRL in breast tissues has long been thought to only encompass the phosphorylation of the residue Y694. However, it is becoming clearer that regulation of this transcription factor is more nuanced. Here, we demonstrate for the first time, phosphorylation of S726 and S780 in human breast carcinoma samples. While both residues are phosphorylated in tumor grades I-III, pS726 increases with tumor grade, and is PRL-responsive. Further, pS726 expression is restricted to luminal breast cancer cell lines and PDXs. Conversely, pS780 is PRL-independent, confirming earlier reports that it is constitutively phosphorylated, and occurs in both luminal breast cancer cell lines and PDX lines, but also in the basal-like breast cancer cell line MDA-MB-436^31^. The constitutive, wide-ranging expression of pS780 suggests that it could result from a non-specific phosphorylation event in breast cancer pathogenesis, however studies performed herein determined that it has a non-redundant role for STAT5a in MCF7 breast cancer characteristics.

It has been assumed that pY694-STAT5a is necessary for most, if not all, PRL-induced gene expression changes. This assumption prior to this study had not been directly assessed by qRT-PCR, or, more globally, RNA-sequencing, chromatin immunoprecipitation (ChIP), or ChIP-sequencing^8,9,54–58^. Rather, pharmacologic inhibition or total knockdown of STAT5a had been the focus of previous work to understand STAT5a transcriptional functions, ignoring the gradation of STAT5a transcriptional function that may be in play in breast cancer and other pathologies^9,54,55^. In this study we performed RNA-sequencing of MCF7 cells expressing single-point phospho-deficient STAT5a mutants treated with PRL in attempts to identify differentially expressed genes and functional pathways for cancer outgrowth. Examination of PRL-regulated DEGs from the WT-STAT5a expressing cells compared to previous studies allowed us to first identify core genes responsive to PRL in this study. Then we were able to examine the activation or inhibition of upstream regulators and downstream functional pathways. While loss of pY694-STAT5a had the largest effect on these functional pathways, loss of either of the pS-sites also had some modulatory effect on these pathways which did not mimic the loss of pY694.

For the first time, we have analyzed the loss of STAT5a serine phosphorylation on breast cancer phenotypic characteristics that fall within the hallmarks of cancer^59,60^. As presented here, the roles for the serine residues of STAT5a are nonredundant, exhibiting different effects on cancer characteristics, and different regulatory roles on the canonical pathway of PRL/STAT5a signaling. Our results indicate a role for STAT5a in establishing and maintaining anchorage-independent growth that may rely on phosphorylation of S780. Expression of Y694F-STAT5a led to a decrease in colony number compared to MCF cells expressing WT-STAT5a. However, the expression of S780A-STA5a in MCF7 rescue cells led to a further decrease in colony number. Therefore, phosphorylation of S780 may contribute to the potential for breast cancer cells to establish viable colonies, indicating a delicate balance for STAT5a regulation. On the other hand, loss of pS726-STAT5a had no effect on colony number or size at the end point for the soft agar assay, which indicates that S726 does not singularly contribute to the overall regulatory effect of STAT5a on anchorage-independent growth. Therefore, it is possible that targeting pS780 on STAT5a would decrease tumorigenicity, leading to better outcomes for patients.

The biologic affects from loss of either phospho-serine residue regarding proliferation and viability were likewise nonredundant. Loss of pS726 decreased PRL-stimulated proliferation in MCF7 cells, and increased the expression of cleaved caspase 3, an effector of apoptosis, in T47D cells. Interestingly, the expression of cleaved caspase 7, another effector molecule of apoptosis, was not increased in MCF7 cells. With the loss of caspase 3, MCF7 cells may be at an advantage when faced with yet another hit to cell viability, i.e. the loss of pS726-STAT5a, compared to T47D cells^61^. Increased apoptosis in T47D cells, on the other hand, illustrates the reliance on intact compensatory mechanisms to evade apoptosis, which is lost with expression of the pS726-STAT5a mutant. Loss of pS780-STAT5a was unremarkable on these characteristics, indicating roles for each phospho-serine residue independent of each other.

We sought to determine the mechanism by which loss of STAT5a phospho-residues affected breast cancer characteristics differentially by examining canonical STAT5a activity, and found 1) the canonical phosphorylation of pY694 is independent of either pS residue, and 2) there is nuclear localized STAT5a with loss of pY694, pS726, or pS780, with altered kinetics for translocation. These results determined that the phospho-serine residues of STAT5a perform roles independent from pY694. Taken together with the RNA-seq data, the STAT5a serine residues appear to regulate functional phenotypes at the transcriptional level.

Accordingly, in silico analysis using TF gene set enrichment analysis was used to establish how these residues alter STAT5a function at the transcriptional level. Our data from this analysis indicates that an array of TF families may be involved as compensation with loss of pY- or pS-STAT5a function. In this study, the SREBP TF family had indicated involvement in both Y694F- and S726A-STAT5a PRL-regulated gene expression changes. As a TF, SREBP has been found to play a critical role in breast cancer migration and invasion, and its involvement in conjunction with the loss of pY694 or pS726 may indicate a role in oncogenic signal switches that confound the loss of STAT5a^62^. Activation of SREBPs are required for growth factor-independent growth and proliferation, which could explain our phenotypic findings with our functional studies. Further, SREBP acts downstream of oncogenic PI3K or K-Ras, and our laboratory recently found that expression of K-Ras is a necessary co-factor for PRLr-mediated oncogenic transformation^45^. This illustrates the complexity of transcription factor activity in breast cancer, and the homeostatic mechanisms that may be at play for oncogenic growth.

Future studies of the loss of these phospho-sites on STAT5a are warranted. With loss of one phospho-site, compensatory mechanisms for recovering STAT5a activity may still be in effect. However, if two or more of these phospho-residues are targeted, we may be able to study how only one remaining phospho-site regulates STAT5a activity. Further studies of how loss of these residues affects STAT5a binding to transcriptional cofactors, or to binding sites on chromatin, will uncover mechanisms responsible for STAT5a activity.

## Methods

### Cell lines and culture

Human breast cancer cell lines (T47D and MCF7), obtained from American Type Culture Collection (ATCC, Manassas VA), were maintained in DMEM (Thermo Fisher Scientific, Waltham, MA) supplemented with 10% fetal bovine serum (FBS) and 100 ug/ml penicillin/streptomycin in a humidified 37 °C incubator with 5% CO_2_. Prior to treating cells with prolactin, complete growth media was replaced with serum-free media consisting of phenol red-free DMEM and 0.1% bovine serum albumin (BSA).

### Prolactin treatment

Human recombinant prolactin (PRL) was a gift from Dr. Anthony Kossiakoff (University of Chicago, Chicago, IL). Following 20–24 h of serum-starvation, PRL was added to cells to a final concentration of 250 ng/ml for indicated time points.

### Western blot analysis

Cells were harvested in radioimmunoprecipitation assay buffer (RIPA) containing 1 × protease inhibitor cocktail (cOmplete, 11,697,498,001) and 1% phosphatase inhibitor cocktail II (P5726-5ML, Sigma-Aldrich, St. Louis, MO). Protein separation was achieved under reducing and denaturing conditions using sodium dodecyl sulfate polyacrylamide gel electrophoresis (SDS-PAGE), transferred to a polyvinylidene difluoride (PVDF) membrane, and immunoblotted with anti-phospho-S726- or anti-phospho-S780-STAT5a antibodies obtained from Abcam (ab128896, ab30649, Cambridge, UK), anti-phospho-Y694-STAT5 antibody (9351S, Cell Signaling Technology, Danvers, MA), anti-STAT5a antibody (sc-1081X, Santa Cruz, Dallas, TX), anti-V5 tag antibody (R96025, Thermo Fisher Scientific), or anti-vinculin antibody (MCA465GA, Bio-Rad, Hercules, CA). HRP conjugated secondary antibodies were obtained from Cell Signaling Technology.

### Transient transfection and harvest for apoptosis assay

MCF7 or T47D cells with endogenous STAT5a KD were transfected using Lipofectamine 3000 (L3000015, Thermo Fisher Scientific). 24 h after transfection, cells were scraped in media, collected, and centrifuged for 5 min at 1000 g. Cells were resuspended in ice-cold PBS to wash off remaining media, and centrifuged. The PBS supernatant was discarded, and cells were then resuspended in RIPA lysis buffer and the WB protocol was followed. Blots were probed with anti-cleaved caspase 3 or anti-cleaved caspase 7 antibody (CC3: 9664T; CC7: 8438S; Cell Signaling Technology).

### Lentiviral production and generation of stable cell lines

STAT5a cDNA was cloned into the pTracer-EF/V5-His mammalian expression vector (v. C, #V88720, Thermo Fisher Scientific) in sequence with the C-terminal V5 tag. Site-directed mutagenesis was performed using either Phusion Site-Directed Mutagenesis kit (for non-GC rich areas of the STAT5a sequence; ie, Y694F and S726A; cat #F541, Thermo Fisher Scientific) or Q5 Site-Directed Mutagenesis kit (for GC rich areas of the STAT5a sequence; ie, S780A; cat #E0552S, New England Biolabs, Ipswich, MA). Construct sequences were confirmed by next generation sequencing (Eurofins Genomics, Louisville, KY). STAT5a-V5 constructs were transfected with 1.5 μg pMDG plasmid and 6 μg pCMVΔR8.91 into HEK293T cells (ATCC) using Lipofectamine 3000. 48 h post-transfection, lentiviral supernatant was collected. MCF-7 cells were infected with lentiviral supernatant supplemented with fresh growth media and 8 μg/ml polybrene by spinfection at 500 g for 2 h at 32 °C. Infected cells were selected by Zeocin (cat #R25001, Thermo Fisher Scientific) 48 h post infection.

### Proliferation

Cells were seeded at a density of 7,500 cells per well in the E-Plate 16. The plate was placed in the xCELLigence Real-Time Cell Analyzer (RTCA; Acea Biosciences, San Diego, CA) for 20–24 h in a humidified incubator at 37 °C with 5% CO_2_, until a Cell Index (CI) between 1–2 was reached. At that point, the experiment was paused and the E-Plate 16 was removed from the apparatus, wells were washed twice with PBS, and 100 μl serum-free media (phenol-free DMEM containing 0.1% BSA) was added to the cells and the E-Plate was placed back in the xCELLigence. After 24 h, the experiment was again paused, and 50 μl of media was replaced with either 50 μl of serum-free media as described above, or with serum-free media containing 2 × prolactin for a final concentration of 250 ng/ml in each well. The plate was placed back on the apparatus and the experiment was resumed for at least 72 h.

### Soft agar colony formation

A base agar layer [0.6% noble agar, 10% serum (v/v)] was established in a 6-well plate. The bottom layer was overlaid with 0.5 mL of top agar solution [0.3% noble agar, 10% serum (v/v)] containing 1.0 × 10^5^ cells in single-cell suspension. Cells were incubated at 37 °C for 2 weeks, and then stained using MTT. 10 images/well were obtained for quantification. Quantification was performed using CellProfiler (cellprofiler.org). 2.5 × 103 µm^2^ was the defined cut-off for colony calling.

### RNA isolation for RNA sequencing (RNA-seq)

Cells were plated on 15 cm tissue culture dishes to about 70% confluency in complete media before incubation in serum-free media for 20–24 h in phenol-free DMEM (Life Technologies) with 0.1% FBS, and subsequently treated with PRL (250 ng/ml in PBS) or PBS control for two hours. After treatment, cells were washed with 37 °C PBS and mRNA was isolated with PureLink™ RNA Mini Kit (cat #12183018A) with on-column DNase treatment (PureLink™ DNase Set, cat#12-185-010, Thermo Fisher Scientific) according to manufacturer’s instructions. Each rescue (EV, WT-, Y694F-, S726A-, or S780A-STAT5a) treated with or without PRL (NO/PRL) was assessed in three independent biological replicates, for a total of 30 RNA samples. RNA purity was determined by spectrophotometry at 260, 270, and 280 nm. RNA integrity number (RIN) was assessed on the Agilent 2200 TapeStation Bioanalyzer (Agilent Technologies), all samples possessed RIN ≥ 9. mRNA library preparation and sequencing were performed the Genomics and Microarray Core at the University of Colorado Anschutz Medical Campus (CU Anschutz). mRNA library was prepared by polyA selection on ≥ 500 ng RNA using the Universal Plus mRNA-Seq Library Preparation Kit (NuGEN) following manufacturer’s protocol. Sequencing was performed on the S4 flow cell platform of the Illumina NovaSEQ6000 System (Illumina, Inc), generating ≥ 50 million 150 bp paired-end (PE) reads per sample. All libraries were prepared and sequenced in a single batch/flow cell lane.

### Bioinformatics analysis

FastQ files were analyzed with FastQC, and read trimming was done with CutAdapt to trim adapter contamination^46,47^. Reads were then aligned to the latest version of the human genome, GRCh38 distributed by Gencode. The primary genome fasta file was downloaded from ftp://ftp.ebi.ac.uk/pub/databases/gencode/Gencode_human/release_29/GRCh38.primary_assembly.genome.fa.gz. The matching gene annotation GTF file was downloaded from ftp://ftp.ebi.ac.uk/pub/databases/gencode/Gencode_human/release_29/gencode.v29.primary_assembly.annotation.gtf.gz. STAR v2.7.3a was used to align the fastq files to the human reference genome^63^. STAR log statistics were compiled with the MultiQC tool, and report that over 90% of reads in each sample were uniquely mapped, with less than 5% reads in each sample being left unmapped. Prior to transcript quantification, the GFFRead utility was used to create a transcriptome fasta file from the main reference genome fasta file^64^. Salmon v0.12.0 utilized the transcript fasta file to quantify each transcript from the STAR-aligned data using “quant” mode and library type set to “ISF”^65^. Salmon output included the raw count estimate, TPM value, and estimated transcript length for each gene. The tximport R package was used to import the Salmon quantifications from each sample into R v3.6.0, identify the official gene symbol, and merge raw count and TPM values into a single matrix file^66,67^. Trimmed BAM files of Chr17:42,287,547-42,311,943 for GRCh38 human genome were generated to confirm point-mutations of the STAT5a mutants in Integrated Genome Browser (IGB, BioViz)^68^. Differential expression analyses were performed in EdgeR using the Galaxy web platform, specifically, raw estimated read counts from Salmon were analyzed using the public server at usegalaxy.org^69–71^. Genes with zero expression in all samples included in the contrasts were removed with a CPM filter > 0.5. Genes were filtered on *p* value of < 0.05, and differentially expressed genes (DEGs) were determined using a cutoff of absolute log fold change > 0.58 (fold change > 1.5). DEGs were determined for the following contrasts: each rescue line PRL vs NO (untreated, ie WT_PRL-WT_NO). Heatmaps for DEG expression were generated using heatmap2 in the Galaxy web platform (Galaxy Version 3.0.1). Galaxy employs the heatmap.2 function from the R gplots package, and clustering was done using the Euclidean distance method and the Complete hierarchical clustering method^72^. Lists of DEGs, with gene identifiers and corresponding expression values (in log fold change) and *p* values, were uploaded into Ingenuity Pathway Analysis software (IPA 2020, Qiagen, www.digitalinsights.qiagen.com/products-overview/discovery-insights-portfolio/analysis-and-visualization/qiagen-ipa/) for pathway predictions. Networks were generated in IPA to depict the analysis of the significant genes identified by RNA-seq and predicted upstream regulators as well as significantly enriched functional pathways. Predicted effects were based on concordance of the RNA-seq gene set differential expression values with the annotated network relationships in the Ingenuity knowledge base. Enrichr analysis (https://maayanlab.cloud/Enrichr/) of the ENCODE gene sets library was performed on significant DEGs for the PRL vs untreated contrast of each STAT5a mutant^51^.

### TMA data

Human breast cancer tissues were obtained from VCU Anatomic Pathology in the form of a TMA. All tissues were stripped of all patient identifiers before use and were therefore anonymous to investigators. The TMA consisted of 47 unmatched breast cancer samples including invasive ductal and lobular carcinoma, and ductal carcinoma in situ (DCIS). Each sample has triplicate cores to mitigate against possible tumor heterogeneity. The presence and integrity of tumor in each sample was confirmed by hematoxylin and eosin (H&E) staining. Samples of the TMA were stained for pY694-, pS726-, pS780-, and total STAT5a following standard IHC protocol using 1:25, 1:100, 1:200, 1:1200 titers for each antibody, respectively^45^. The TMA was scored independently by a clinical pathologist in the VCU Department of Pathology and scored for both nuclear staining proportion score and intensity score for Allred score calculations^73^.

### Statistical analysis

Statistical analysis was performed using one-way or two-way analysis in GraphPad Prism 9.0.0 for Windows, GraphPad Software, San Diego, California USA, www.graphpad.com. Results are reported as means ± SEM (standard error of the mean). *p* < 0.05 was considered statistically significant. All experiments were done three times unless otherwise noted.

## Supplementary Information

Supplementary Information.

## Data Availability

All data generated and analyzed in this study are included in this published article. RNA-seq datasets have been submitted to the National Center for Biotechnology Information Gene Expression Omnibus (GEO) and are accessible through GEO Series Accession Number GSE165678 (https://www.ncbi.nlm.nih.gov/geo/query/acc.cgi?acc = GSE165678).
