# Supplementary material for: Serine residues 726 and 780 have nonredundant roles regulating STAT5a activity in luminal breast cancer
Source: Sci Rep. 2021 Jun 29;11:13506. doi: 10.1038/s41598-021-92830-8 (PMC8242097; doi:10.1038/s41598-021-92830-8)

Title: Serine residues 726 and 780 have nonredundant roles regulating STAT5a activity in luminal breast cancer

Authors: Alicia E. Woock, Jacqueline M. Grible, Amy L. Olex, J. Chuck Harrell, Patricija Zot, Michael Idowu, Charles V. Clevenger

Supplementary Figure Legends

Supplementary Figure S1. TMA for pY694-STAT5a shows weak nuclear staining. Representative anti-pY694 and anti-STAT5a IHC images from unmatched Grade III, ER-/PR-/Her2 amplified primary tumors included in the TMA (scale bars, 100 µm). Graphs show quantification of modified Nuclear Allred scores according to the tumor grade, Ki67 status, and molecular subtypes.

Supplementary Figure S2. Confirmation of STAT5a knockdown and rescue at the mRNA level in MCF7 cells. MCF7 cells with stable knockdown (KD) and rescue of STAT5a by indicated constructs analyzed by qRT-PCR for STAT5a mRNA. **p<0.001, ****p<0.0001.

Supplementary Figure S3. Loss of STAT5a phospho-sites differentially affect PRL-regulated gene expression patterns. Hierarchical clustering of the significantly differentially expressed genes from RNA-seq analysis of PRL-treated versus untreated MCF7 cells expressing STAT5a or one of the STAT5a phospho-mutants.

Supplementary Figure S4. Gene induction is affected differentially by loss of STAT5a phosphorylation. A) Expression log ratios of select PRL-inducible or repressed genes from the RNA-sequencing visualized in IPA. B) Expression log ratios of the same panel of PRL-induced DEGs of each mutant STAT5a compared to WT-STAT5a + PRL DEGs visualized in IPA, illustrating how each mutant specifically increases or decreases expression of these genes compared to WT-STAT5a + PRL. C)-F) Confirmatory qRT-PCR analysis of the same panel of PRL-inducible or PRL-repressed genes, normalized to total STAT5a expression and GAPDH for each sample. **p=0.005, ***p<0.0001 vs. WT-STAT5a + PRL. #p<0.00005 rescue PRL vs. untreated.

Supplementary Figure S5. Effect of single-point phospho-deficient STAT5a mutants on characteristics of MCF7 cells. A) Representative micrographs of anchorage-independent growth assayed by soft agar for WT-STAT5a, EV, or S726A-STAT5a at endpoint. B) Scratch wound assay for migration, *$p \leq 0.04$, ***$p = 0.008$ compared to WT-STAT5a.

Supplementary Figure S6. Validation of specificity of STAT5a staining using empty vector (EV) control cell line. MCF7 cells transduced with EV pTracer were stained with anti-STAT5a antibody and imaged as in **Figure 6**. Representative merged Hoechst and α-STAT5a images from time course illustrate lack of STAT5a staining.

Supplementary Figure S1

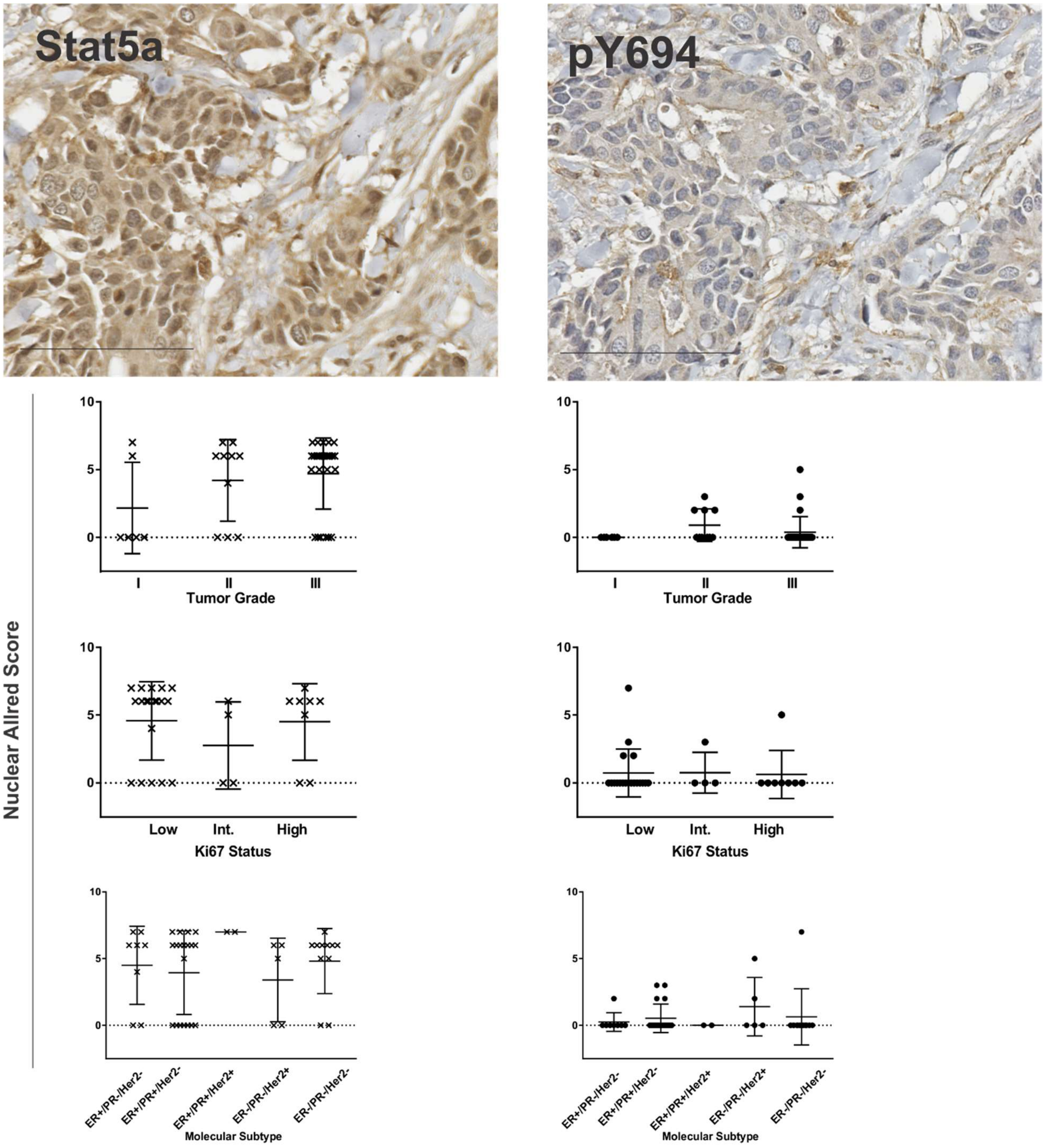

Supplementary Figure S2

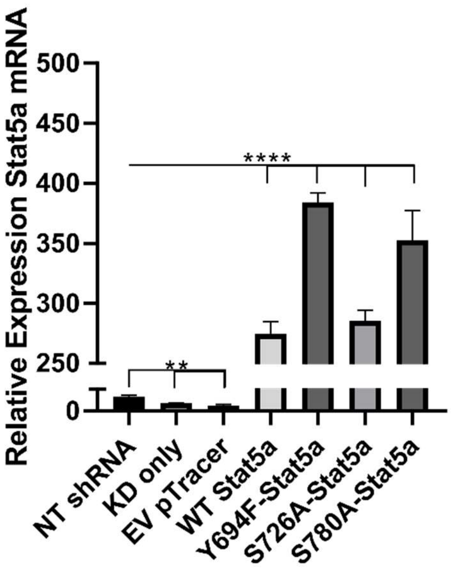

Supplementary Figure S3

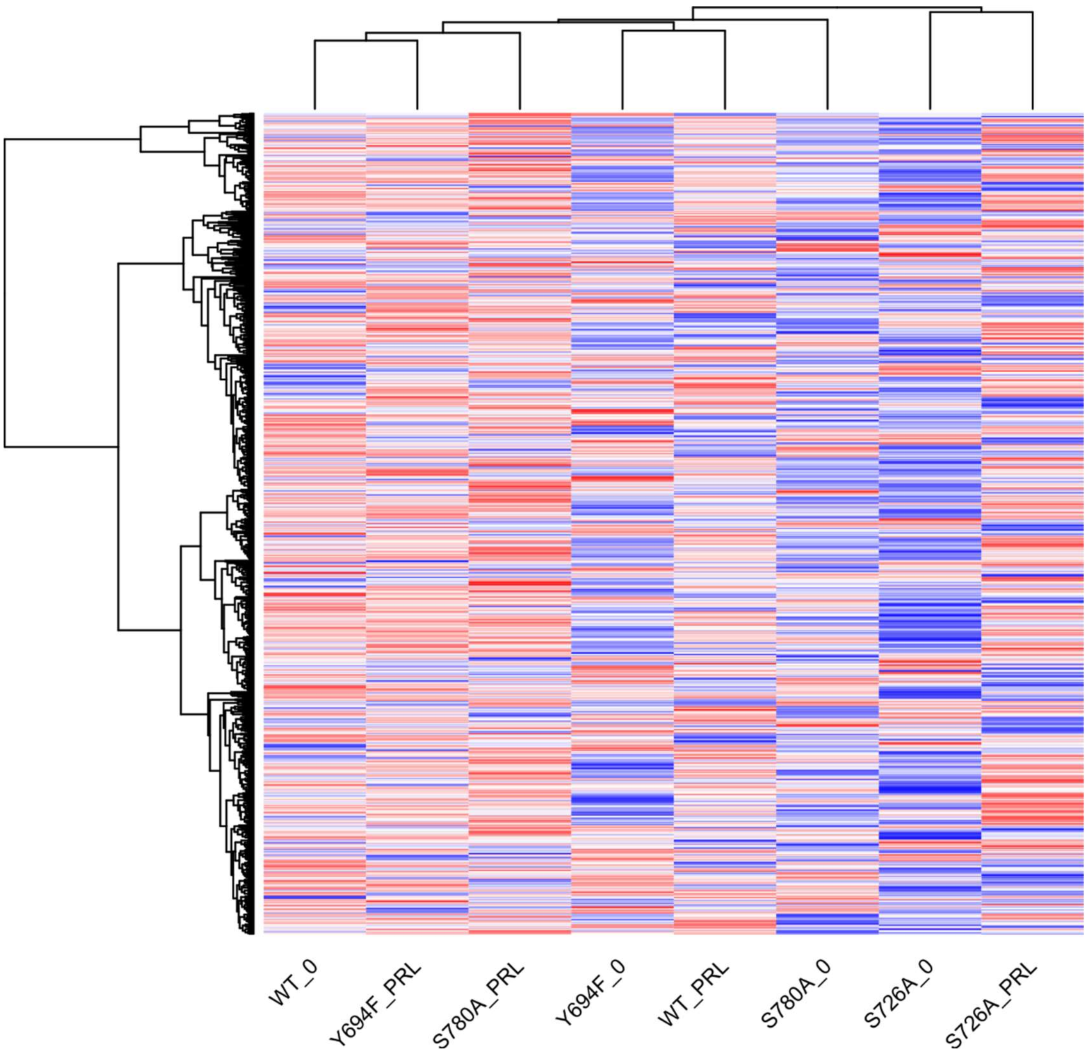

Supplementary Figure S4

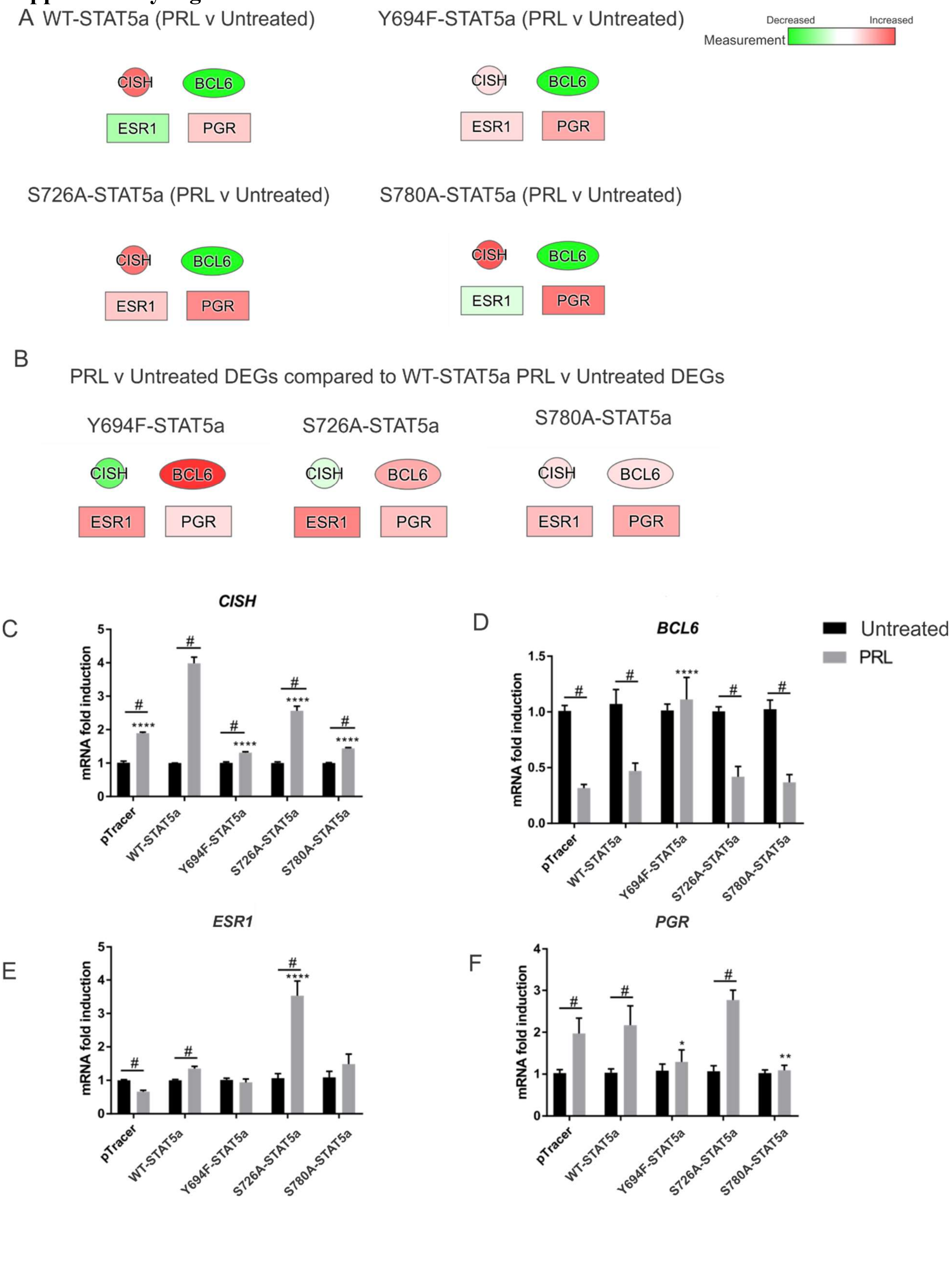

Supplementary Figure S5

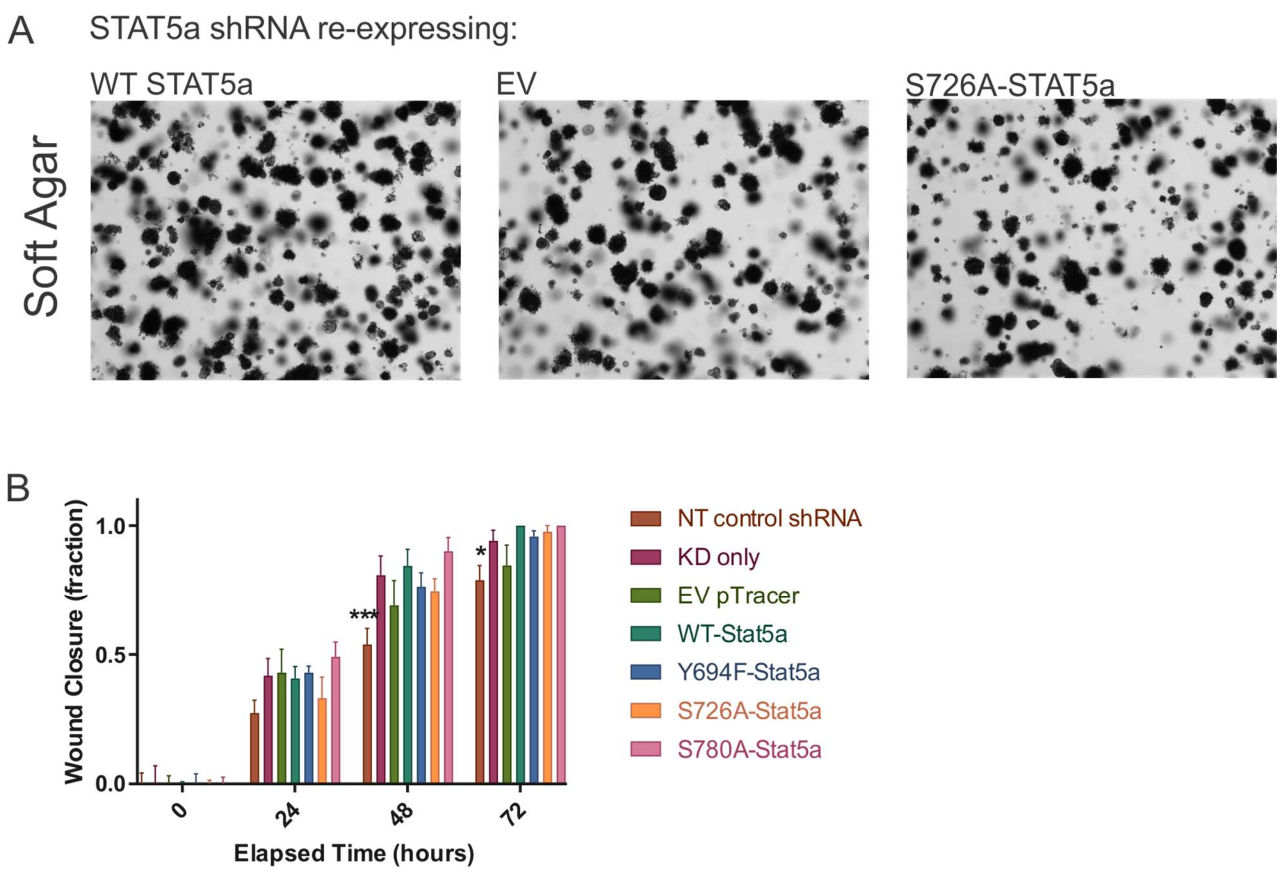

Supplementary Figure S6

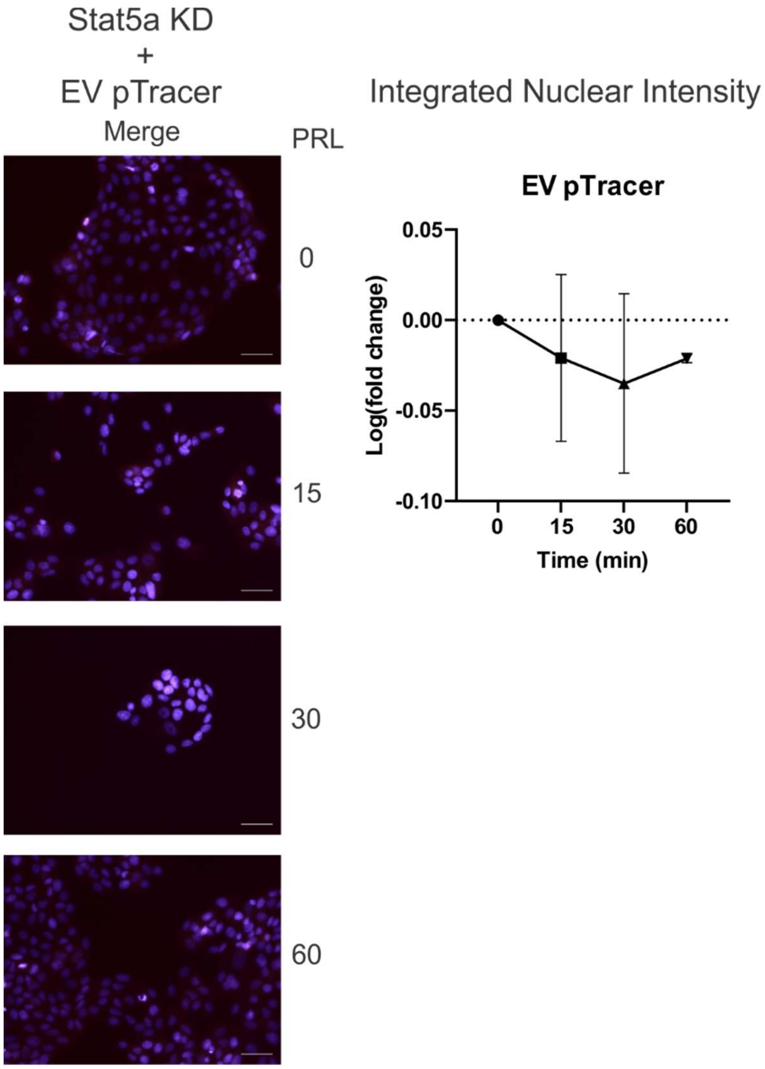

Supplementary Table S1. Patient characteristics of TMA.

Characteristic	TMA (N=47)
**Age-yr.**	
Median	55
Range	25-90
**Race-n (%)**	
Black	27 (57.4)
Hispanic	1 (2.1)
White	17 (36.2)
Other	2 (4.3)
**Histologic Grade-n (%)**	
I	6 (12.8)
II	10 (21.3)
III	27 (57.4)
Not reported	4 (8.5)
**Ki67 status-n (%)**	
Low	18 (38.3)
Intermediate	4 (8.5)
High	8 (17.0)
Not reported	16 (34.0)
**ER Status-n (%)**	
Negative	16 (34.0)
Positive	31 (65.9)
**PR Status-n (%)**	
Negative	26 (55.3)
Positive	21 (44.7)
**HER2 amplified Status-n (%)**	
Negative	38 (80.9)
Positive	9 (19.1)
**Hormone Receptor Status-n (%)**	
ER+/ PR- /Her2-	8 (17.0)
ER+ /PR+/ Her2-	19 (40.4)
+/+/+	2 (4.3)
ER- /PR- /Her2+	5 (10.6)
-/-/-	11 (23.4)
ER+ /PR- /Her2+	2 (4.3)

Supplementary Table S2. Molecular subtypes and expression of hormone receptors and STAT5a in breast cancer patient derived xenografts (PDX) and cell lines.

	**ER**	**PR**	**HER2**	**STAT5a**	**Molecular Subtype**
**PDX**					
**HCI-011**	+	+	-	+	**Luminal B**
**HCI-008**	-	-	+	+	**HER2-enriched**
**HCI-001**	-	-	-	+	**Basal-like**
**HCI-009**	-	-	-	-	**Luminal**
**WHIM2**	-	-	-	+	**Basal-like**
**WHIM30**	-	-	-	+	**Basal-like**
**Cell line**					
**T47D**	+	+	-	+	**Luminal A/B**
**MCF7**	+	+	-	+	**Luminal A/B**
**BT-474**	+	+	+	+	**HER2-enriched**
**SUM1315**	-	-	-	-	**Basal-like**
**MDA-MB-231**	-	-	-	+	**Claudin-low**
**MDA-MB-436**	-	-	-	+	**Claudin-low**

Supplementary Table S3. Differentially expressed genes determined by RNA-seq analysis of STAT5a-rescued MCF7 cells treated with PRL.

**Contrast: PRL v 0**	**Upregulated Genes**	**Downregulated Genes**
**WT-STAT5a**	235	275
**Y694F-STAT5a**	90	81
**S726A-STAT5a**	178	107
**S780A-STAT5a**	182	26

Supplementary Table S4. Quality check on WT-STAT5a (PRL vs untreated) dataset presented compared to microarray/RNA seq published previously.

	Fiorillo et al[38]		Hakim et al[39]		Craig et al[52]		Woock et al	
Gene	FC	p value	FC	p value	logFC	p value	logFC	p value
BCL6	-3.088	0.00828	-3.085	1.29E-19	-2.267	7.58E-10	-2.511	7.23E-86
HBP1	-1.72	0.00261	-0.61	1.15E-14	-0.567	0.000266	-0.327	0.000568
FBXO32	-1.577	0.0192			-0.293	0.000216	-0.467	0.000171
YPEL2	-1.544	0.0324					-0.323	0.00322
TSC22D3	-1.543	0.0281	-0.743	7.63E-16	-0.54	5.96E-07		
TXNIP	-1.489	0.047	-0.615	1.39E-13	-0.528	3.51E-06		
ZNF627	-1.422	0.0275					-0.39	0.00221
FAM217B	-1.417	0.0171					-0.25	0.0159
EFNA1	-1.398	0.0336	-0.524	9.55E-11	-0.218	0.00086		
PAQR6	-1.359	0.00055	-0.203	0.00624	0.339	0.000841		
TTLL3	-1.287	0.0388	-0.22	0.00731	0.43	0.000623		
NPHP3	-1.264	0.0259					-0.447	0.006
FRAT1	-1.203	0.00489	-0.691	2.03E-12	-0.412	0.000263		
PYCR3	1.222	0.019	0.165	0.00408			0.207	0.0242
JMJD6	1.238	0.0137	0.737	1.23E-11	0.446	5.07E-05	0.327	0.00774
STX1A	1.242	0.00406					0.295	0.0308
SPHK1	1.243	0.0389	0.426	5.52E-09	0.366	0.00106	0.217	0.0151
CHKA	1.25	0.0143	0.327	0.000156	0.27	0.000416		
OSGIN1	1.252	0.0349	0.458	4.16E-08	0.485	0.00106	0.339	0.000842
ANKRD27	1.265	0.0341	0.236	3.55E-06			-0.255	0.00613
SENP5	1.273	0.0269	0.39	6.66E-08			-0.226	0.0395
GCNT1	1.275	0.0414	0.667	2.67E-08			0.601	0.000347
ICOSLG/LOC102723996	1.287	0.00244			0.662	4.5E-07	0.589	2.31E-06
TRMT61A	1.291	0.0143	0.177	0.00433			0.212	0.0325
ETS2	1.309	0.00607	0.696	1.64E-10			0.239	0.0247
CD3EAP	1.313	0.00169	0.428	3.31E-08	0.346	0.000262	0.403	0.000142
RPP25	1.314	0.0465	0.301	4.81E-06			0.307	0.00261
ASB7	1.333	0.0016	0.202	0.000531			-0.28	0.0359
USP36	1.333	0.00442	0.849	8.59E-08	0.441	6.78E-06	0.229	0.01
PLA2G15	1.337	0.0162	0.362	1.24E-06	0.34	0.00115	0.225	0.0477
RRP1	1.337	0.0299	0.458	1.58E-08			0.265	0.00356
PPAN	1.346	0.00442	0.447	9.98E-10	0.44	0.000804	0.388	5.23E-05
PDGFB	1.348	0.0148	0.217	0.00404			0.223	0.0178
LRRC8E	1.361	0.00953	0.479	4.07E-08			0.245	0.031
ZNF324	1.368	0.0344	0.203	0.000122			0.33	0.0012
ABCF2	1.369	0.045	0.338	1.91E-07			0.204	0.0226
G6PD	1.371	0.0355					0.177	0.0446
TXNRD1	1.383	0.0388	0.119	0.000864			-0.196	0.0232
MINPP1	1.385	0.0498	0.234	0.000107			-0.297	0.0407
PA2G4	1.387	0.0295	0.109	0.00489			0.197	0.021
DDX28	1.405	0.0111	0.16	0.0036			0.309	0.00676
RCL1	1.411	0.0167	0.411	2E-09	0.407	1.11E-05	0.392	0.000643
KLF4	1.415	0.00312	0.392	0.000122			-0.262	0.005

TMEM158	1.415	0.000137	0.497	0.000588			0.904	0.00435
ADRM1	1.419	0.0315	0.225	4.72E-06			0.181	0.0372
DDX56	1.432	0.0457	0.147	0.00476			0.212	0.0155
TSPAN14	1.437	0.0264	0.158	0.00176			0.237	0.0058
MAT2A	1.437	0.0271	0.625	4.87E-14	0.331	4.06E-05	0.276	0.00133
CISH	1.44	0.00202	0.707	2.04E-11	1.318	2.96E-11	0.645	7.74E-10
RRP7A	1.45	0.0445	0.224	0.00011	0.331	0.000269	0.218	0.0201
CSRP1	1.466	0.0151	0.322	7.19E-08			0.226	0.00835
NOTCH1	1.471	0.00367	0.473	3.21E-07			1.381	0.000584
JMJD4	1.476	0.000247	0.338	1.2E-06			0.234	0.0304
DHCR7	1.487	0.0144	0.172	0.000741			0.201	0.0187
PUS1	1.495	0.0423	0.395	2.88E-09	0.372	0.000418	0.238	0.0141
GRPEL1	1.515	0.0248	0.565	3.44E-14			0.25	0.0109
MIR22HG	1.516	0.000606	0.892	6.15E-14			0.357	0.0464
NOP56	1.526	0.00315	0.306	0.000061			0.229	0.0101
SLC20A1	1.531	0.0126	0.46	3.49E-09			0.264	0.00748
DOLK	1.533	0.004	0.155	0.00355			0.286	0.00328
BYSL	1.556	0.0126	0.515	7.16E-13	0.451	5.61E-05	0.332	0.00291
RCAN1	1.572	0.00661	0.57	1.38E-07	0.771	3.2E-07	0.667	1.17E-09
CCDC86	1.584	0.0107	0.42	4.51E-11	0.428	5.74E-05	0.34	0.00111
POLR1C	1.584	0.0011	0.438	1.65E-11	0.467	2.82E-05	0.31	0.00628
GRWD1	1.589	0.0423	0.252	4.79E-05			0.221	0.0152
RNF126	1.589	0.017	0.39	2.58E-06			0.231	0.0208
TWNK	1.602	0.00806	0.449	8.96E-07	0.305	0.00012	0.232	0.0232
CEBPA	1.628	0.0234	0.585	1.88E-07			0.314	0.00204
MSRB1	1.638	0.016	0.374	3.51E-10	0.395	0.000237	0.431	2.33E-06
NOP2	1.644	0.0166	0.349	1.07E-06	0.289	0.000286	0.186	0.04
AUNIP	1.649	0.0107	0.408	2.46E-07			0.376	0.00931
GEMIN4	1.671	0.0345	0.37	3.9E-07	0.262	0.000329	0.263	0.00396
SLC2A1	1.678	0.0272	0.202	0.000548			0.226	0.00818
BRPF1	1.707	0.00448	0.347	8.76E-09			0.218	0.0163
CNN2	1.711	0.00473	0.267	9.19E-05			0.171	0.047
NOL6	1.722	0.0314	0.272	9.44E-05			0.215	0.0174
NXT1	1.775	0.00217	0.433	3.4E-11	0.359	0.000282	0.25	0.0252
NOP16	1.817	0.000846	0.641	3.69E-12	0.478	4.15E-05	0.294	0.00239
AEN	1.829	0.0206	0.516	9.69E-11	0.36	9.44E-06	0.309	0.00185
FJX1	1.885	0.000274	0.538	9.96E-11			0.309	0.0112
RIOX1	1.906	0.0022	0.251	2.32E-05			0.236	0.0148
CEBPB	1.932	0.000818	0.804	5.82E-17			0.264	0.00347
PPRC1	2.007	0.000593	0.53	1.31E-09	0.462	2.56E-05	0.333	0.000193
RRS1	2.053	0.00289	0.359	8.47E-10	0.23	0.000894	0.263	0.00412
ZYX	2.076	0.000448	0.854	9.08E-11	0.511	1.67E-05	0.186	0.0308
RARA	2.166	0.000791	0.838	4.18E-10	0.639	4.77E-08	0.436	4.1E-07
SPATA2L	2.326	0.00281	0.556	1.48E-09	0.423	0.00105	0.294	0.00309
TUFT1	2.351	0.000781	1.259	1.85E-17			0.215	0.0278
PHLDA2	2.384	0.000907	1.187	4.65E-13	0.699	3.23E-06	0.952	4.1E-08
IER3	2.417	0.000551	0.777	8.23E-13	0.444	5.27E-07	0.382	4.08E-05
TNFRSF12A	3.04	8.74E-05	1.331	2.09E-10	0.443	1.12E-05	0.259	0.00255

MYADM	3.398	5.61E-07			0.352	0.000167	0.366	0.000041
BTG1			-0.729	6.27E-13	-0.467	0.000066	-0.19	0.0282
NR2F2			-0.545	1.27E-06	-0.495	0.00095	-0.289	0.000869
STARD13			-0.543	1.96E-09	-0.556	0.000314	-0.292	0.00877
ACKR3			-0.538	5.04E-08	-0.606	0.000585	-0.238	0.00694
TSC22D1			-0.534	1.6E-12	-0.516	0.000148	-0.256	0.005
CDKN1B			-0.491	2.67E-08	-0.661	4.44E-05	-0.303	0.00103
CITED2			-0.465	2.48E-06	-0.575	0.000143	-0.325	0.000159
NAB2			0.206	0.000994	0.417	4.74E-07	0.289	0.00104
PNP			0.317	5.32E-10	0.327	6.27E-05	0.228	0.0303
MRTO4			0.317	8.66E-05	0.327	0.000624	0.253	0.00729
MYBBP1A			0.347	7.32E-06	0.278	5.17E-05	0.198	0.0232
LOC102724159/PWP2			0.472	2.66E-10	0.329	8.06E-05	0.216	0.0441
JUN			1.466	3.43E-14	0.301	0.00102	0.207	0.0403

Supplementary Table S5. Primers for qRT-PCR

Gene Target	For and Rev primers (5'-3')
BCL6	For CTGCAGATGGAGCATGTTGT
	Rev TCTTCACGAGGAGGCTTGAT
CISH	For AGAGGAGGATCTGCTGTGCAT
	Rev GGAACCCCAATACCAGCCAG
ESR1	For GGAAGCTACTGTTTGCTCCTAACTTG
	Rev AGATCTCCACCATGCCCTCTAC
GAPDH	For CATGAGAAGTATGACAACAGCCT
	Rev AGTCCTTCCACGATACCAAAGT
PGR	For TCAGTGGGCAGATGCTGTATTT
	Rev GCCACATGGTAAGGCATAATGA
STAT5a	For GTTCAGTGTTGGCAGCAATGAGC
	Rev AGCACAGTAGCCGTGGCATTGT

Supplementary Methods

qRT-PCR (Supplementary Figure S3)

Total RNA was isolated using TRI Reagent Solution (AM9738, Thermo Fisher Scientific) in an RNase-free environment. cDNA was synthesized using the iScript cDNA synthesis kit (1708891, Bio-Rad) following manufacturer's protocol and 1 μg of purified RNA as the template for the reaction. For qRT-PCR, 100 ng of the first strand synthesis reaction was used as a template with 900 nM each of forward and reverse primers in 1x Power SYBR Green PCR Master Mix (43-685-77, Thermo Fisher Scientific). Primers are listed in Supplemental Table S5. Each reaction was run in triplicate in a Hard-Shell Clear 384-well reaction plate in a CFX384 thermal cycler (Bio-rad). Data were normalized to total Stat5a mRNA, and fold change was calculated using the comparative ΔΔCt method.

Wound closure assay (Supplementary Figure S4)

The Incucyte WoundMaker (Sartorius, Essen Biosciences, Ann Arbor, MI) was used to create wounds in a 96 well plate. Following manufacturer's protocol, breast cancer cells were seeded at a density of 40,000 cells per well. Cells adhered to the plate at 37°C for 20-24 h. A scratch was made in all wells simultaneously following manufacturer's protocol. After washing, complete media was added to the wells before placing the plate in the Incucyte. Image acquisition was programmed 10x every 3 hours. Image data were analyzed using Incucyte software and quantification of wound width closure over time was performed in Excel.

Expanded Data Supplementary File with Full Western Blots

Figure 1B: Full membrane western blot data

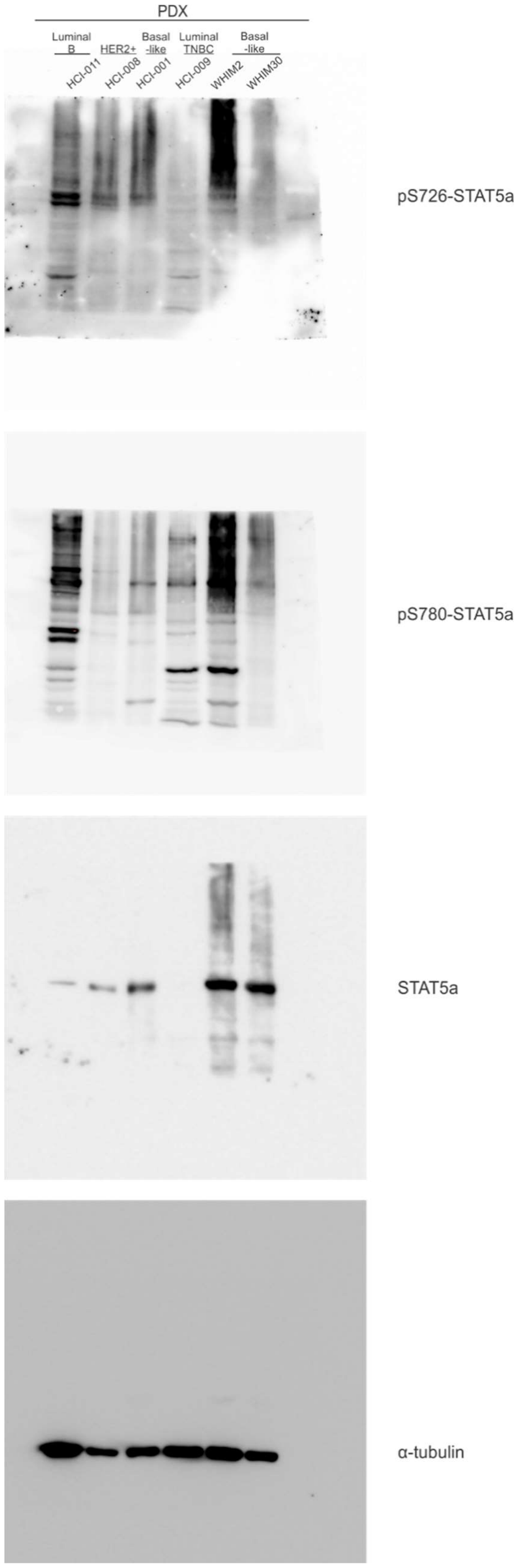

Figure 1C: Full membrane western blot data

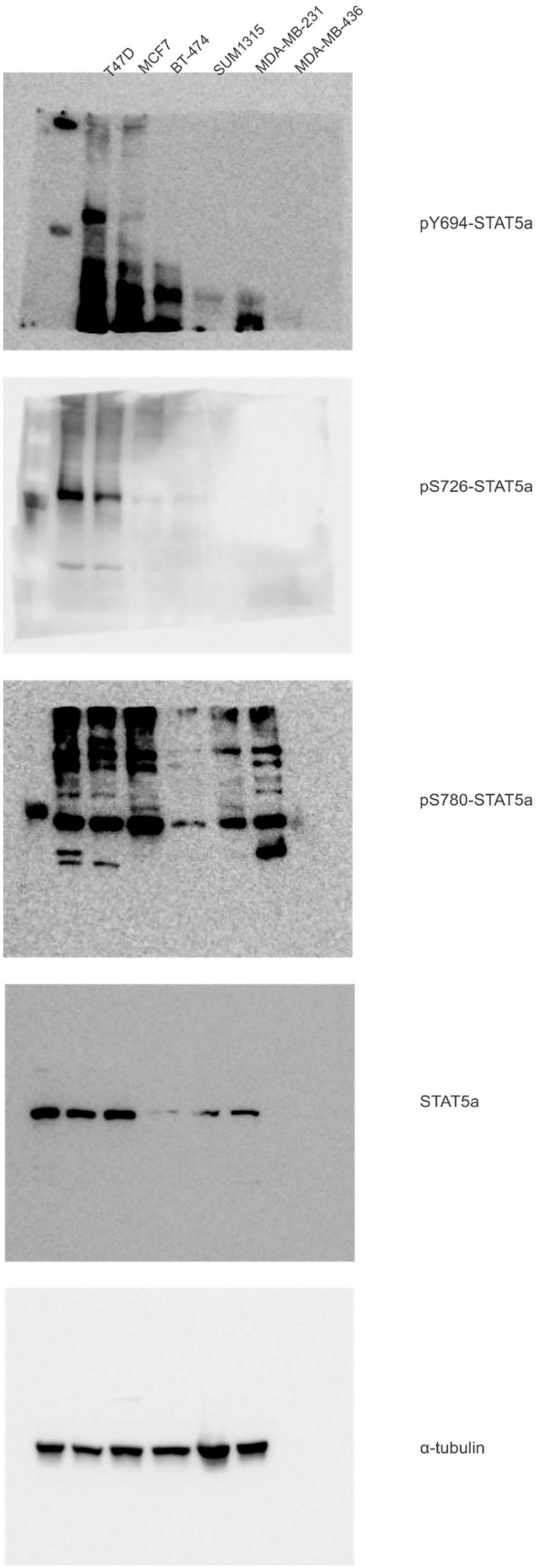

Figure 1D: Full membrane western blot data

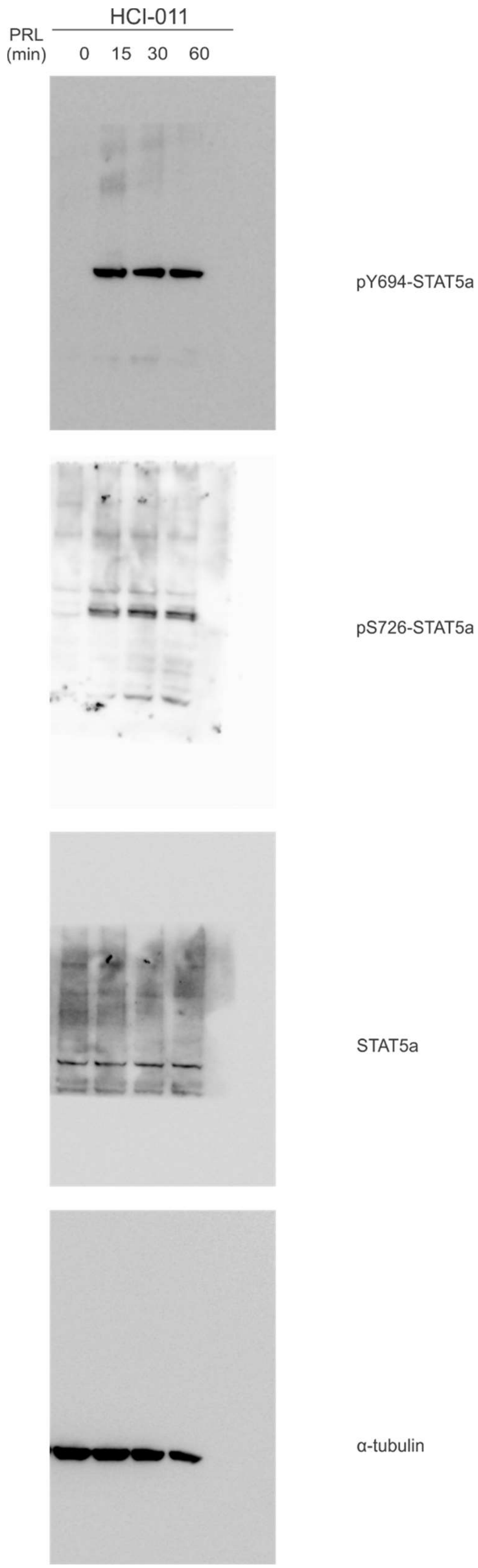

Figure 1E: Full membrane western blot data

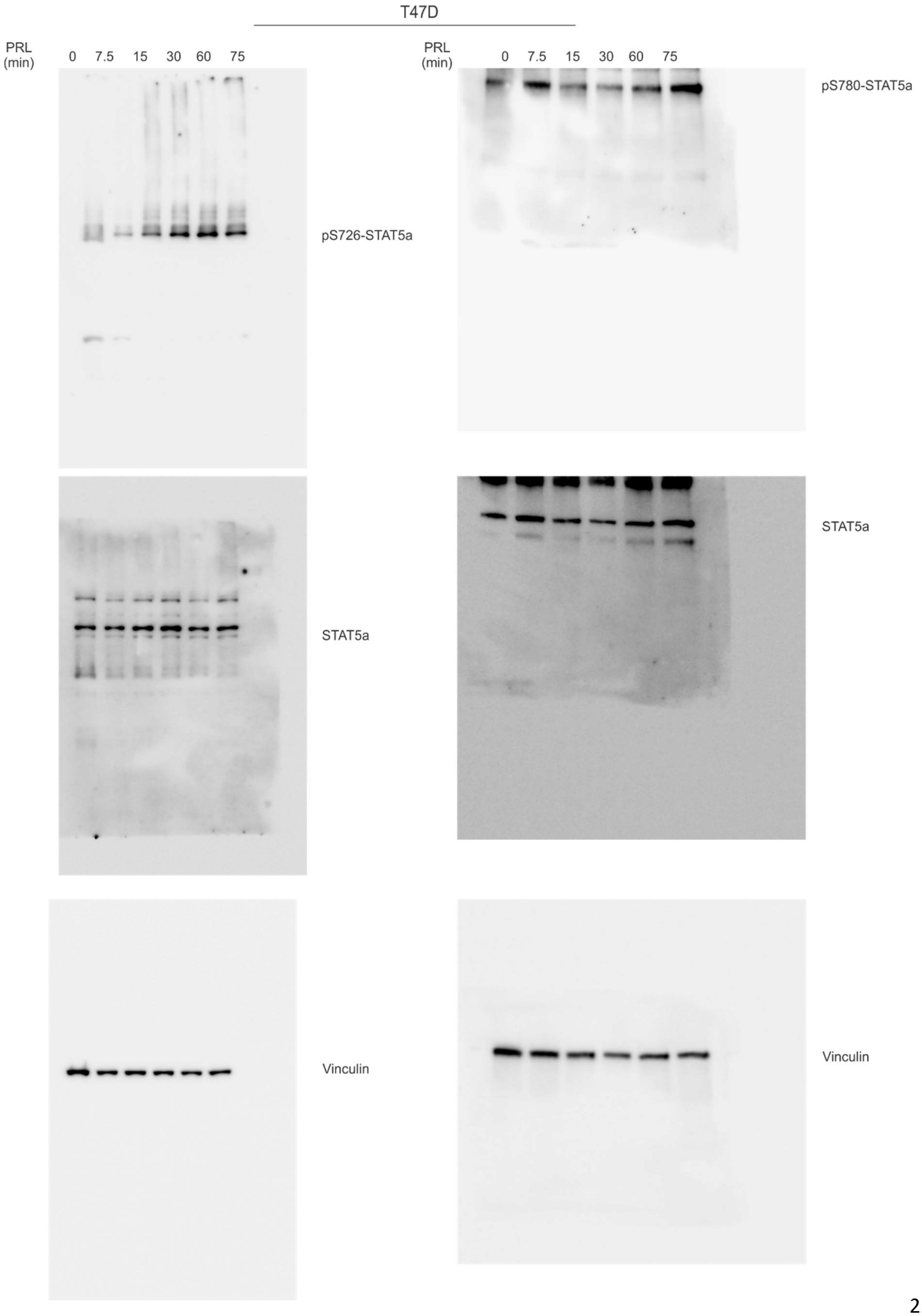

Figure 1F: Full membrane western blot data

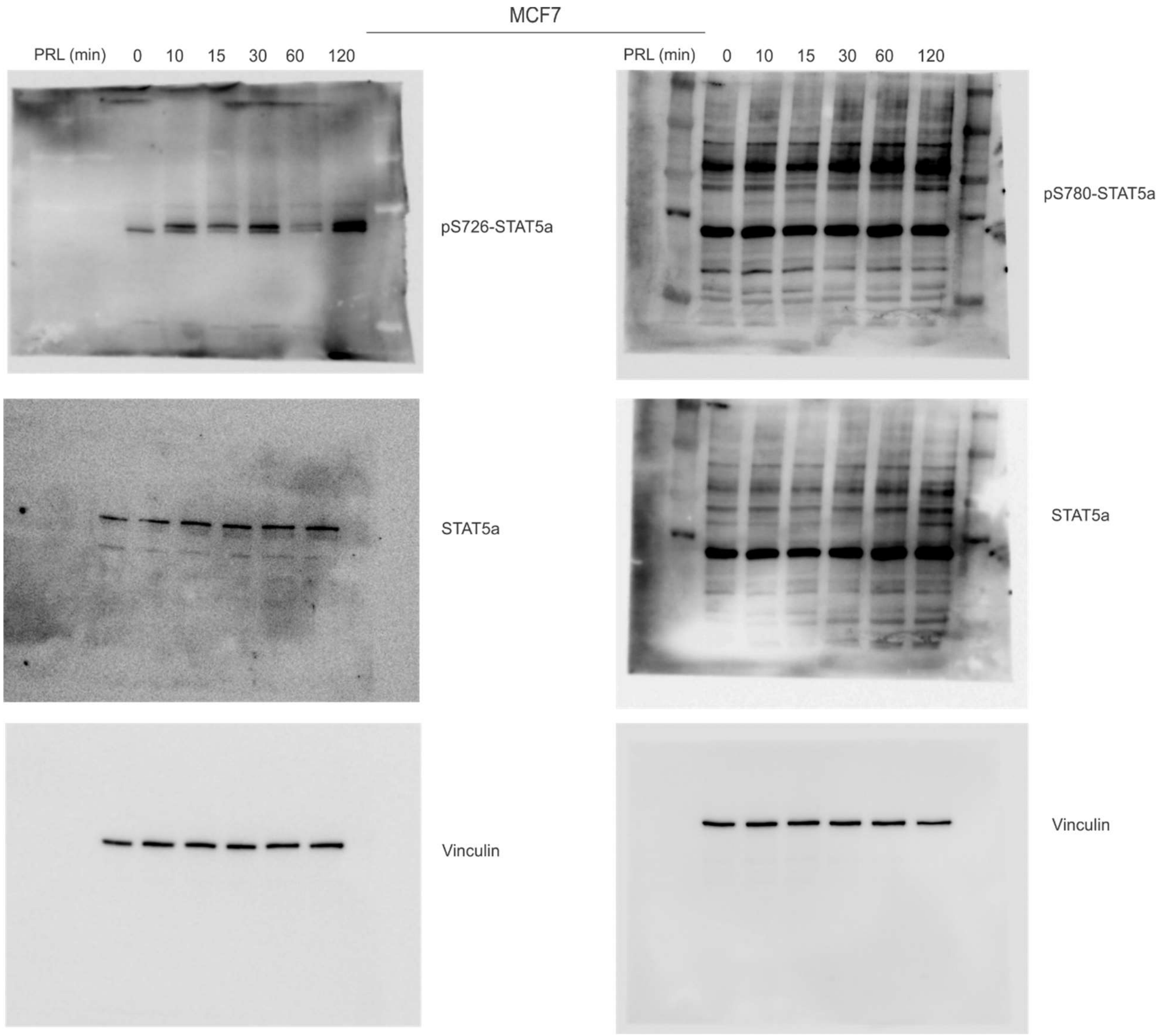

Figure 2B: Full membrane western blot data

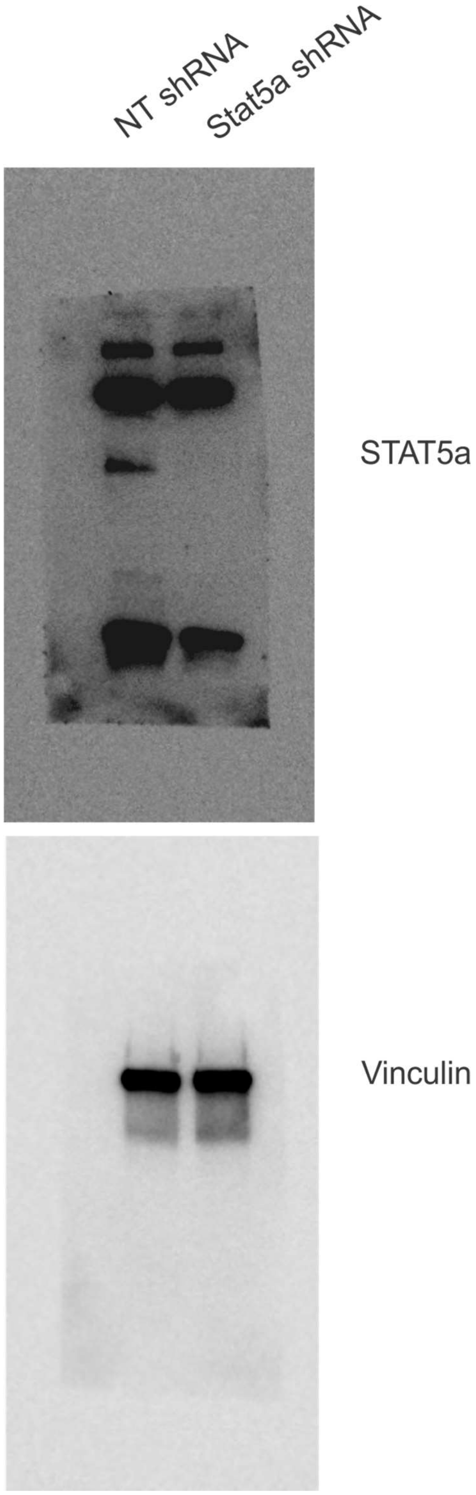

Figure 2D: Full membrane western blot data

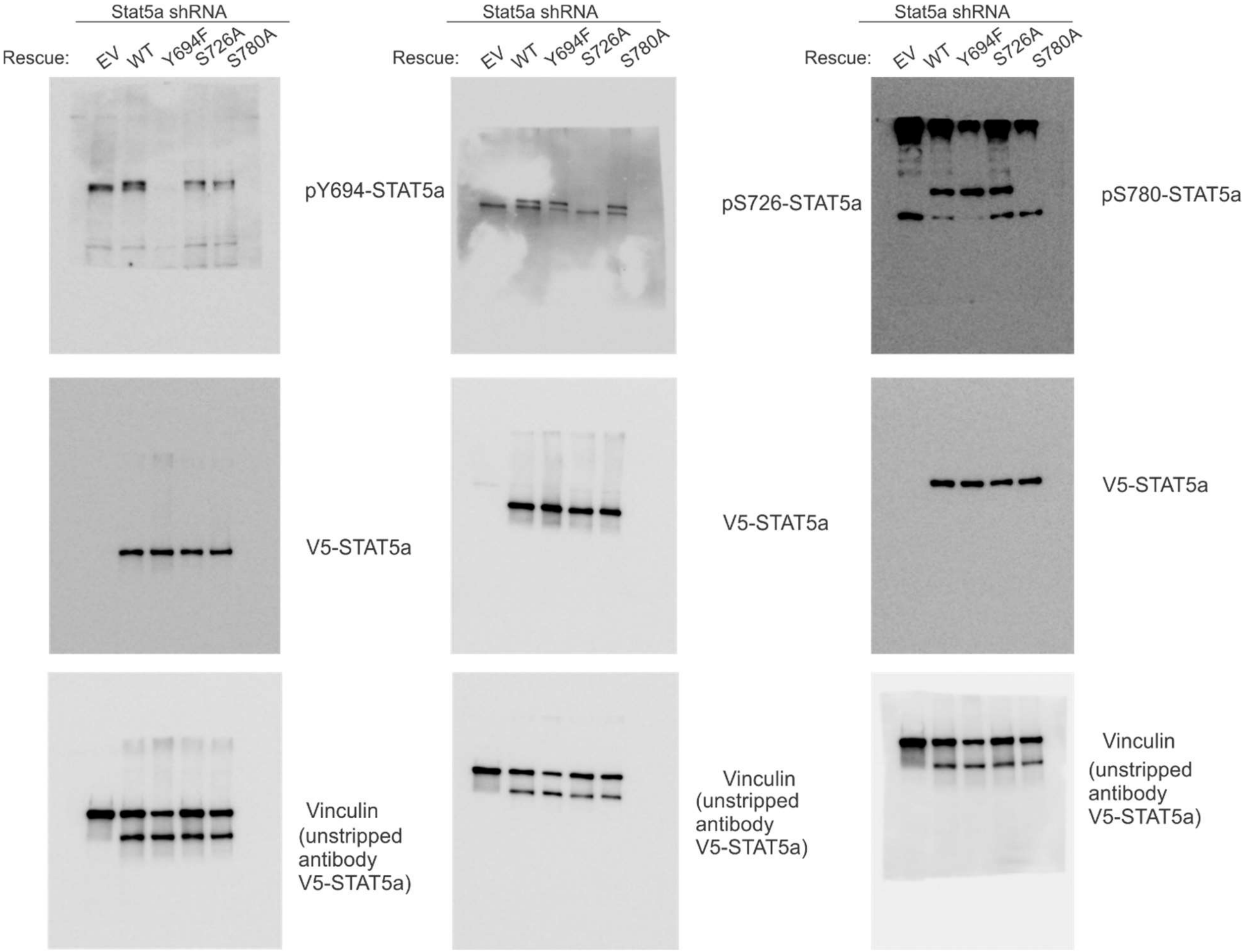

Figure 4C: Full membrane western blot data

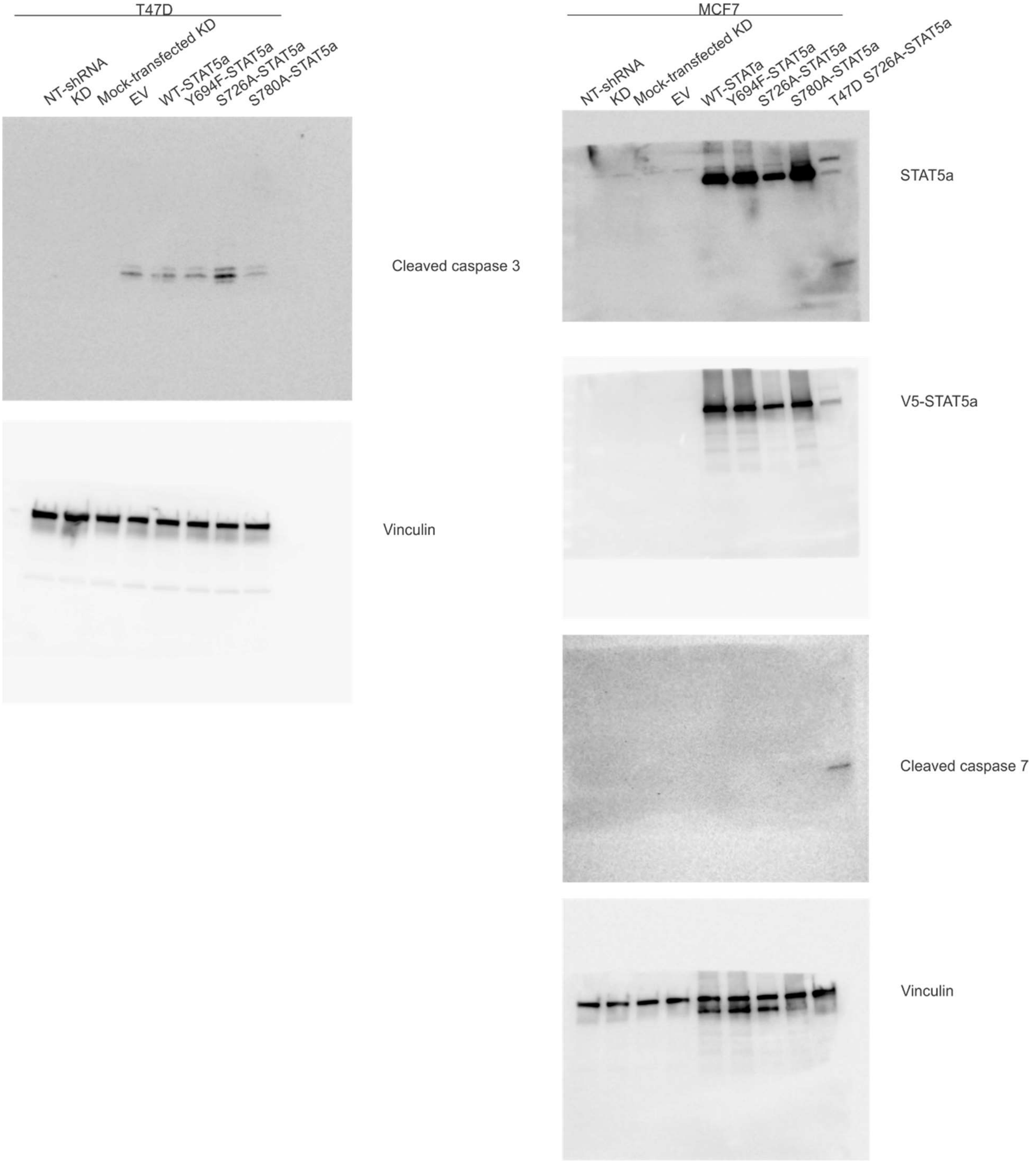

Figure 5A and 5B: Full membrane western blot data

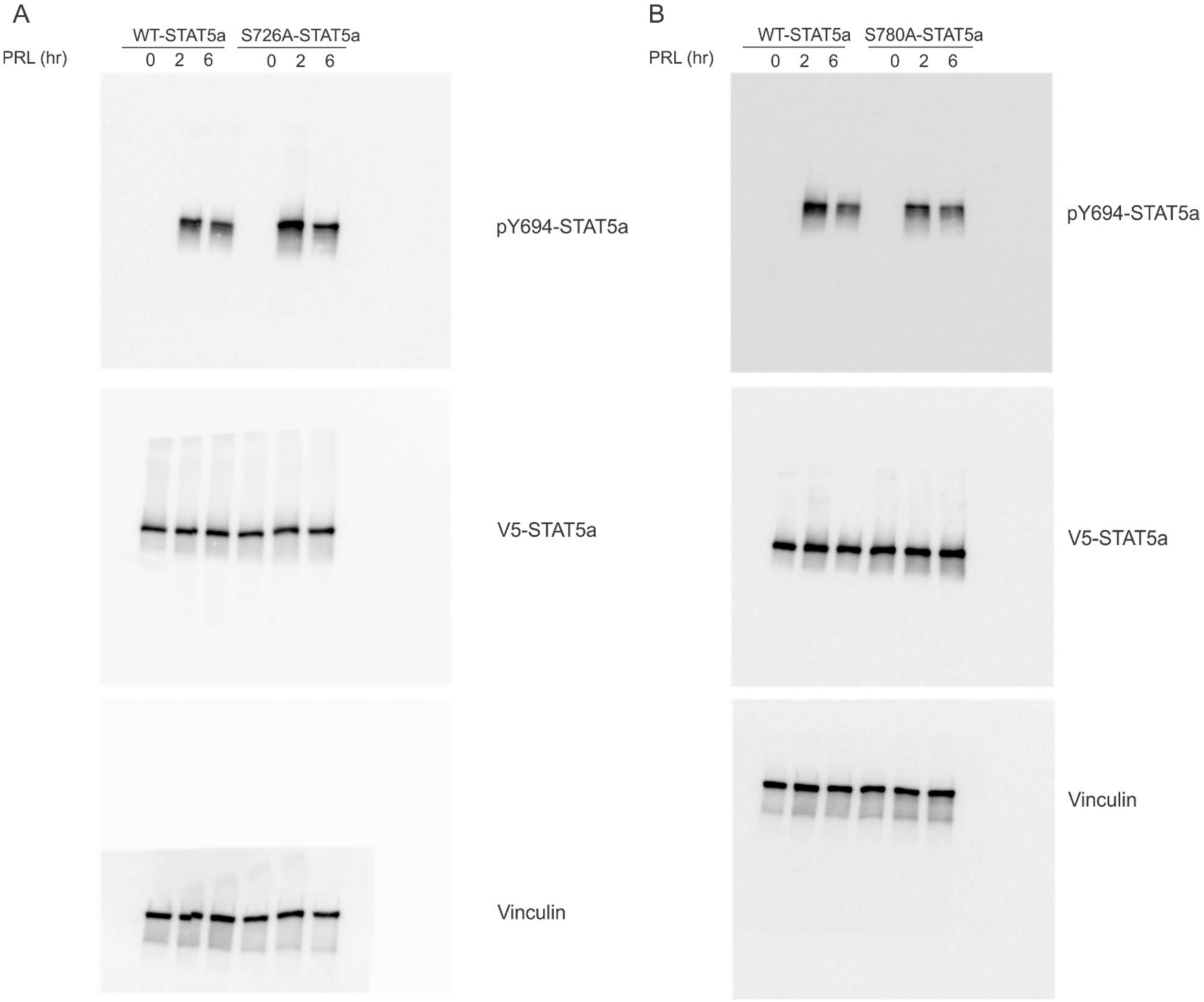

Figure 6B: Full membrane western blot data

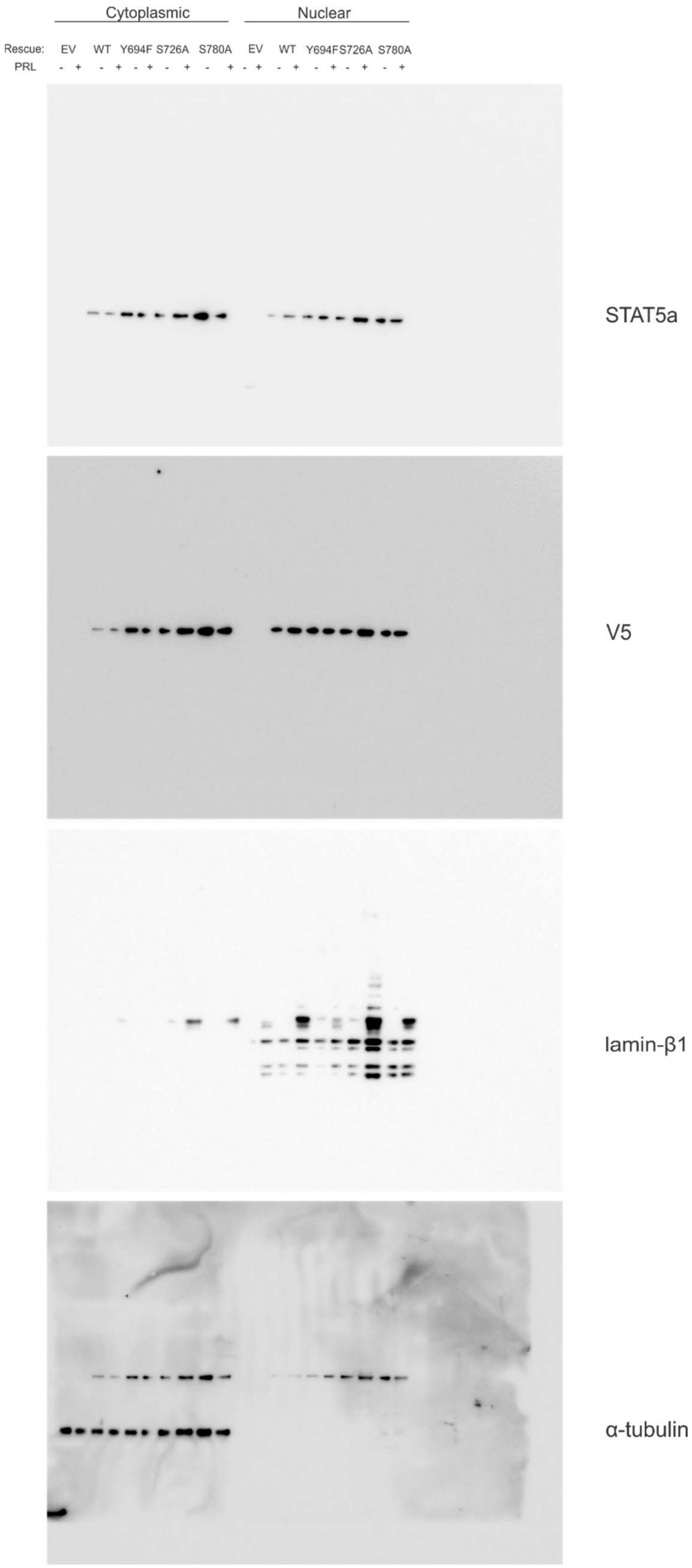

Supplement: Supplementary file 1 — Supplementary Information. [file 41598_2021_92830_MOESM1_ESM.pdf]
